# Genetic variations of low-density lipoprotein cholesterol on metabolic disorders in obstructive sleep apnea

**DOI:** 10.1186/s12986-024-00805-z

**Published:** 2024-06-10

**Authors:** Yu Peng, Hangdong Shen, Chenyang Li, Xiaoyue Zhu, Yiqing Gao, Hongliang Yi, Huajun Xu, Jian Guan, Xinyi Li, Shankai Yin

**Affiliations:** 1 Department of Otorhinolaryngology Head and Neck Surgery, Shanghai Key Laboratory of Sleep Disordered Breathing, Shanghai Sixth People’s Hospital Affiliated to Shanghai Jiao Tong University School of Medicine&, 600 Yishan Road, Shanghai, 200233 P. R. China; 2 Otolaryngology Institute of Shanghai Jiao Tong University, Shanghai, China

**Keywords:** Low-density lipoprotein cholesterol, Obstructive sleep apnea, Cardiovascular diseases, Insulin resistance, Metabolic syndrome, Single nucleotide polymorphism

## Abstract

**Background:**

The study aimed to explore the relationship between low-density lipoprotein cholesterol (LDL-C) genetic variants and obstructive sleep apnea (OSA) and its complications, including cardiovascular diseases (CVD), insulin resistance (IR), and metabolic syndrome (MS).

**Method:**

4329 individuals with suspected OSA who underwent a comprehensive assessment of anthropometric, biochemical, and polysomnography (PSG) data, along with 30 LDL-C single nucleotide polymorphisms (SNPs) were enrolled. The 10-year Framingham CVD risk score (FRS), IR and MS were evaluated for each subject. Linear regression and logistic regression were utilized to examine the correlations among these variables.

**Results:**

After the Benjamini-Hochberg correction, linear regression results indicated positive correlations between variants rs3741297 and rs629301 with FRS (β = 0.031, P_BH_=0.002; β = 0.026, P_BH_=0.015). Logistic regression revealed that rs3741297 increased MS risk among total subjects [OR = 1.67 (95% CI:1.369–2.038), P_BH_=1.32 × 10^− 5^] and increased IR risk in females [OR = 3.475 (95% CI:1.653–7.307), P_BH_=0.03]. In males, rs2642438 decreased MS risk [OR = 0.81 (95% CI:0.703–0.933), P_BH_=0.045].

**Conclusions:**

The rs3741297 variant correlated with susceptibility to CVD, IR, and MS in the OSA population. OSA, CVD, IR and MS share a potentially common genetic background, which may promote precision medicine.

**Cinical trial registration:**

The study protocol was registered with the Chinese Clinical Trial Registry (ChiCTR1900025714).

**Supplementary Information:**

The online version contains supplementary material available at 10.1186/s12986-024-00805-z.

## Introduction

Obstructive Sleep Apnea (OSA), a prevalent sleep breathing disorder, affects 9–38% of the general population [1]. In recent years, the incidence of OSA has rapidly increased, drawing global attention. It is estimated that approximately 936 million people worldwide suffer from OSA, with China holding the foremost position [2]. Owing to metabolic disruptions induced by nocturnal hypoxia and fragmented sleep, individuals with OSA emerge as a pivotal demographic for the prevention and treatment of cardiovascular diseases (CVD), insulin resistance (IR), and metabolic syndrome (MS) [3–5].

OSA frequently coexists with abnormal levels of low-density lipoprotein cholesterol (LDL-C), potentially resulting in a cascade of metabolic consequences [6]. The elevated levels of LDL-C may account for the heightened risk of atherosclerosis in the OSA population [7]. This association is supported by another study, indicating a 1.42 times relative increase in the risk of CVD due to the interaction between amplified LDL-C levels and OSA [8]. Additionally, increased LDL-C levels disrupt glucose metabolism, raising the risk of IR in individuals with OSA [9]. Recent evidence also suggests an enhanced susceptibility to MS with augmented LDL-C elevation [10]. However, it remains unclear whether elevated LDL-C levels act as intermediate factors between OSA and its complications or result from the interaction between OSA and its complications. Some studies propose that heightened LDL-C levels manifest when OSA is combined with IR [11, 12], while the association between OSA itself and LDL-C levels is confounded by factors such as gender and obesity [11, 13].

LDL-C levels are influenced by various factors, making it challenging to eliminate confounding variables in experiments. Conversely, genetic variations associated with LDL-C exhibit stability, facilitating bias control and yielding more reliable results. Twin-based research has revealed a shared genetic foundation linking OSA and dyslipidemia [14]. Common complications of OSA, such as CVD, IR, and MS, are also subject to genetic influence [15–17]. A nuanced examination of the association between LDL-C genetic variations and these diseases may provide an innovative perspective. Recent advancements in Genome-Wide Association Studies (GWAS) have unearthed numerous single nucleotide polymorphism (SNP) loci affecting LDL-C levels. Our study aimed to explore the correlation between LDL-C SNPs and OSA, CVD, IR, and MS. Considering the impact of ethnicity on genetic variations, we intentionally sourced SNP data from GWAS conducted on East Asian populations [18, 19].

## Methods

### Subjects

A total of 5635 individuals with suspected OSA were enrolled in the ongoing Shanghai Sleep Health Study (SSHS) (previously detailed in [20]). Following their informed consent, medical history questionnaires, anthropometric measurements, polysomnography (PSG) data, biochemical profiles, and SNP information were collected. Subjects meeting specific exclusion criteria were omitted from the study: (1) those with missing PSG data (*n* = 473); (2) those lacking LDL-C data (*n* = 361); (3) those with more than 10% missing SNP data (*n* = 388); (4) individuals with prior OSA-related treatments such as continuous positive pressure ventilation or upper airway surgery (*n* = 12); (5) frequent users of lipid-lowering medications (*n* = 44); (6) those afflicted with severe systemic diseases including chronic heart, liver, lung, or kidney failure (*n* = 8); and (7) individuals with severe mental disorders or other sleep disorders like central sleep apnea or episodic somnolence (*n* = 20). Ultimately, 4329 participants were approved for analysis by the Institutional Ethics Committee of the Sixth People’s Hospital affiliated with Shanghai Jiao Tong University, as depicted in Figure S1.

### SNP selection

Three genome-wide genotyping platforms were utilized: Affymetrix Genome-Wide Human SNP Array 6.0 (SNP6.0), Affymetrix Axiom Genome-Wide CHB1 Array Plate and Illumina 1 M Array. The genotyping procedures, quality control measures, and genotype imputation of Chinese data were described in our GWAS [21]. In this study, 64 SNPs associated with LDL-C levels were sourced from East Asian population-based GWAS (*P < 5 × 10*^*− 8*^) [18, 19]. Rigorous quality control, executed through PLINK software (v1.90), led to the exclusion of SNP loci based on the criteria such as missing data exceeding 10% of the total samples (*n* = 18), deviation from Hardy-Weinberg equilibrium (*P* < 0.05) (*n* = 4), minor allele frequency below 0.01 (*n* = 3), and linkage disequilibrium (r2 > 0.2) with other variants in the same genomic region (*n* = 9). Following this stringent quality control process, 30 SNPs met the criteria and were considered suitable for subsequent investigation, as depicted in Figure S2. The exploration of the relationship between these SNPs and gene expression involved correlations established through expression quantitative trait loci (eQTL) analysis using the 3DSNP database.

### Anthropometry and biochemistry

Healthcare professionals adhered to standardized protocols for anthropometric assessments: Weight and height were measured on barefoot individuals wearing lightweight attire, with body mass index (BMI) computed as weight divided by height squared. Waist circumference (WC) was measured in the middle of the lowest rib margin and the iliac crest. Hip circumference (HC) was measured at the widest point of the buttocks. Systolic (SBP) and diastolic blood pressure (DBP) were derived from an average of three readings using an Omron Model HEM-752 Fuzzy device following a 10-minute rest period. After PSG, fasting blood samples were collected the subsequent morning and subjected to analysis for fasting blood glucose (FBG), total cholesterol (TC), triglycerides (TG), high-density lipoprotein cholesterol (HDL-C), and LDL-C levels utilizing an automated analyzer (H-7600; Hitachi, Tokyo, Japan). Serum fasting insulin levels were determined via immunoassay techniques.

According to the World Health Organization’s standards, participants who had smoked continuously or cumulatively for six months or more were defined as smokers [22]. Men consuming over 60 g and women consuming over 40 g of pure alcohol daily were defined as drinkers [23]. Individual 10-year Framingham CVD risk score (FRS) was computed based on age, HDL-C and TC levels, SBP, usage of antihypertensive medication, documented diagnosis of diabetes mellitus (DM), and smoking status [24]. Participants were divided into two groups based on their FRS: those with FRS > 20% were classified as high-risk for CVD, while the rest were classified as intermediate-low risk for CVD. Individual Homeostasis Model Assessment of Insulin Resistance (HOMA-IR) was determined by multiplying fasting insulin levels (µIU/mL) by FBG levels (mmol/L) and dividing by 22.5 [25]. An individual was considered IR if their HOMA-IR ≥ 2.5. MS was defined as the presence of three or more of the following criteria [26]: (1) male WC ≥ 90 cm or female WC ≥ 80 cm; (2) SBP ≥ 130 mmHg, DBP ≥ 85 mmHg, or a documented diagnosis of hypertension; (3) FBG ≥ 100 mg /dL or individuals receiving medication for Type 2 DM; (4) TG ≥ 150 mg/dL; (5) HDL-C < 40 mg /dL for men or < 50 mg /dL for women.

### PSG assessment and definition

Trained sleep technicians assessed sleep metrics using PSG equipment (Alice 4 or 5; Respironics, Pittsburgh, PA, United States) following the 2012 American Academy of Sleep Medicine (AASM) criteria [27]: Apnea was defined as a ≥ 90% reduction in oro-nasal airflow lasting ≥ 10 s, and hypoventilation was defined as a ≥ 30% deviation for ≥ 10 s accompanied by a ≥ 3% reduction in oxygen saturation or sleep arousal. The apnea-hypopnea index (AHI), calculated by averaging apnea and hypoventilation events per hour of sleep, was utilized to classify the severity of OSA: non-OSA (< 5.0), mild (5.0-14.9), moderate (15.0-29.9), and severe (≥ 30.0). The oxygen desaturation index (ODI) quantified episodes of ≥ 3% oxygen desaturation per hour of sleep. Cumulative time percentage with SpO2 < 90% (CT90) indicated the percentage of sleep time spent with oxygen saturation levels below 90%. Lowest oxygen saturation (LSpO2) represented the lowest recorded oxygen saturation level. The micro-arousal index (MAI) computed the average number of awakenings per hour of sleep.

### Statistical analysis

SPSS software (version 19.0, IBM Corp, Armonk, NY, USA) was employed for statistical analyses. Descriptive statistics were utilized to present normally distributed data as mean and standard deviation, skewed data as median and interquartile range, and categorical data as frequencies and percentages. Baseline characteristics among groups were compared using the ANOVA test, the Kruskal-Wallis test, or the χ2-test based on data distributions. Multivariate linear regression was conducted to investigate the associations between individual SNPs and PSG data (AHI, ODI, CT90, LSpO2, and MAI), as well as metabolic indicators (FRS, glucose, insulin, and HOMA-IR). Subsequently, binary logistic regression was performed to investigate the correlations between individual SNPs and CVD, IR, and MS. Linear and logistic regressions were performed under an additive genetic model, adjusting for confounding variables including age, gender, BMI, smoking, and alcohol intake. Results were presented as (β, P) for linear regression and [OR (95%), P] for logistic regression. A two-tailed *P* value < 0.05 was considered statistically significant. To correct for multiple testing, the Benjamini–Hochberg (BH) method was performed, which applied a false discovery rate (FDR). The P-values from regression analyses of 30 SNP variants were ranked (n = 1 to 30), and adjusted using the formula P_BH n_=P_n_× 30 / n. Starting from P_BH 30_, if P_BH n−1_> P_BH n_, the formula was skipped, setting P_BH n−1 _ equal to P_BH n _until P_BH 1 _value was reached. A two-tailed P_BH _value < 0.05 was considered to pass the multiple testing.

## Results

### Baseline

The study encompassed a total of 4,329 subjects, exhibiting a mean age of 43.4 (± 12.6) years old and a mean BMI of 27.2 (± 4.1) kg/m2, with males constituting 88% of the cohort. In Table 1, subjects were stratified into distinct categories based on their AHI: no (549), mild (256), moderate (938), and severe (2585) OSA groups. The severe OSA cohort displayed notably elevated levels of LDL-C, TC, and TG, alongside an increased susceptibility to CVD, IR and MS when compared to the other categorized groups. The details of the SNPs were listed in table S1, presenting information regarding respective genes, major alleles, minor alleles, minor allele frequencies, as well as chromosomal locations and positions. Furthermore, the results of linear regression analyses for these SNPs concerning subjects’ levels of LDL-C, HDL-C, TC, and TG were comprehensively outlined in Tables S2 to S5.

**Table 1:** Basic characteristics of the enrolled subjects classified by OSA severity

Variable	Non-OSA (*n* = 549)	Mild OSA (*n* = 256)	Moderate OSA (*n* = 938)	Severe OSA (*n* = 2585)	*P*
Demographics					
age (years)	38(26–50)	32(27–37)	46(33–59)	45(33–57)	< 0.001
Male (%)	549(100%)	256(100%)	741(79.0%)	2275(88.0%)	< 0.001
BMI (kg/m2)	24.4(21.0–27.8)	25.3(22.4–28.2)	26.5(22.6–30.3)	28.3(24.2–32.3)	< 0.001
WC (cm)	89(80–99)	92(84–100)	95(86–105)	101(90–111)	< 0.001
HC (cm)	98(91–104)	100(94–105)	101(94–109)	104(96–112)	< 0.001
Biochemistry assays					
Glucose (mmol/L)	5.2(4.17–6.23)	5.03(4.56–5.5)	5.57(4.28–6.86)	5.74(4.52–6.96)	< 0.001
Insulin (uU/mL)	8.99(3.66–14.32)	10.81(4.28–17.34)	12.69(4.39–20.99)	15.32(6.08–24.56)	< 0.001
HOMA-IR	2.17(0.49–3.85)	2.45(0.82–4.08)	3.2(0.62–5.78)	3.98(1.10–6.86)	< 0.001
TC (mmol/L)	4.42(3.54–5.3)	4.57(3.69–5.45)	4.78(3.86–5.7)	4.89(3.94–5.84)	< 0.001
TG (mmol/L)	1.58(0.34–2.82)	1.67(-0.03–3.37)	1.97(0.39–3.55)	2.27(0.36–4.18)	< 0.001
HDL-C (mmol/L)	1.08(0.83–1.33)	1.09(0.86–1.32)	1.07(0.83–1.31)	1.03(0.81–1.25)	< 0.001
LDL-C (mmol/L)	2.76(2.01–3.51)	2.93(2.13–3.73)	3.03(2.22–3.84)	3.05(2.26–3.84)	< 0.001
SBP (mmHg)	121(107–136)	123(110–135)	126(110–143)	130(114–146)	< 0.001
DBP (mmHg)	77(68–87)	77(67–88)	80(69–91)	83(71–95)	< 0.001
Sleep apnea					
AHI	2.2(0.8–3.7)	10.7(8.3–13.0)	22.1(17.8–26.3)	58.1(40.8–75.3)	< 0.001
ODI	5.6(-10.2–21.4)	11.3(4.8–17.8)	24.0(13.1–34.9)	58.1(37.2–79.0)	< 0.001
CT90(%)	0.6(-4.1–5.2)	1.2(-3.3–5.8)	3.9(-3.0–10.7)	20.2(1.8–38.6)	< 0.001
LSpO2(%)	92(86–98)	86(80–92)	81(73–90)	70(57–82)	< 0.001
MAI	18.0(4.4–31.5)	21.4(6.0–36.8)	24.1(7.6–40.6)	37.4(14.7–60.1)	< 0.001
Medical history					
Smoker (%)	117(18.0%)	90(35.2%)	159(17.0%)	439(17.0%)	< 0.001
Drinker (%)	280(51.0%)	84(32.8%)	460(49.0%)	1448(56.0%)	< 0.001
FRS	5.18(-0.22–10.58)	3.99(1.23–6.75)	8.75(3.18–14.32)	9.32(3.81–14.83)	< 0.001
high CVD risk (%)	44(8.0%)	3(1.2%)	122(13.0%)	439(17.0%)	< 0.001
IR (%)	159(29.0%)	92(35.9%)	488(52.0%)	1706(66.0%)	< 0.001
MS (%)	159(29.0%)	82(32.0%)	460(49.0%)	1706(66.0%)	< 0.001

The normally distributed data are presented as the means and standard deviation; skewed data are presented as the median (IQR), and categorical data are presented as the number (percentage). Differences in the baseline characteristics among the four groups were examined using different methods based on the data distributions. The Kruskal-Wallis test was used for differences in TC, CT90, and LSpO2, the χ2-test was used for differences in gender, smoking, alcohol consumption, high CVD risk, IR, and MS, and the ANOVA test was used for differences in other variables

BMI, body mass index; WC, waist circumference; HC, hip circumference; HOMA-IR, homeostasis model assessment for insulin resistance; TC, total cholesterol; TG, triglyceride; HDL-C, high-density lipoprotein cholesterol; LDL-C, low-density lipoprotein cholesterol; SBP, systolic blood pressure, DBP, diastolic blood pressure; AHI, apnea–hypopnea index; ODI, oxygen desaturation index; CT90, cumulative time percentage with SpO2 < 90%; LSpO2, lowest oxygen saturation; MAI, micro-arousal index; FRS, the general 10-year Framingham CVD risk score; IR, insulin resistance; MS, metabolic syndrome

### SNP and OSA

A thorough investigation into the associations between the enrolled SNPs and PSG parameters (AHI, ODI, CT90, LSpO2, MAI, respectively) was conducted using multiple linear regression analyses, as presented in Tables S6-S10. Subsequently, pertinent results were extracted and consolidated in Table 2. The rs7780562 variant exhibited positive associations with both AHI and ODI (β = 0.036, *P* = 0.01; β = 0.037, *P* = 0.008; respectively). Conversely, the rs76898656 variant exhibited negative correlations with both AHI and CT90 in male subjects (β=-0.032, *P* = 0.024; β=-0.031, *P* = 0.039; respectively). In addition, the rs2954027 variant displayed a negative correlation with ODI (β=-0.028, *P* = 0.044), and a positive correlation with LSpO2 (β = 0.028, *P* = 0.047). Both the rs10987829 and rs2419607 variants manifested positive correlations with LSpO2 (β = 0.028, *P* = 0.044; β = 0.029, *P* = 0.043; respectively). The rs7140110 variant positively aligned with MAI (β = 0.04, *P* = 0.008), while the rs7412 variant exhibited a negative association with MAI (β=-0.035, *P* = 0.019). Moreover, rs2539981 displayed a trend towards correlation with decreased MAI (β=-0.029, *P* = 0.05). However, none of these SNPs were statistically significantly associated with PSG parameters after BH correction.

**Table 2:** Significant results in linear regression of enrolled SNPs and PSG parameters

	SNP	total			men			women		
		β	*P*	*P*_BH_	β	*P*	*P*_BH_	β	*P*	*P*_BH_
AHI	rs7780562	0.036	**0.010**	0.300	0.028	0.054	0.438	0.074	0.083	0.959
	rs76898656	-0.025	0.068	0.630	-0.032	**0.024**	0.438	0.032	0.447	0.959
ODI	rs7780562	0.037	**0.008**	0.240	0.032	**0.031**	0.690	0.051	0.233	0.981
	rs2954027	-0.028	**0.044**	0.660	-0.023	0.115	0.690	-0.057	0.180	0.981
CT90	rs76898656	-0.027	0.059	0.591	-0.031	**0.039**	0.785	-0.002	0.962	0.987
LSpO2	rs2954027	0.028	**0.047**	0.470	0.02	0.180	0.613	0.087	0.038	0.390
	rs10987829	0.028	**0.044**	0.470	0.033	**0.028**	0.613	0	0.991	0.991
	rs2419607	0.029	**0.043**	0.470	0.029	0.055	0.613	0.038	0.373	0.979
MAI	rs2539981	-0.029	0.050	0.500	-0.025	0.118	0.968	-0.073	0.093	0.626
	rs7140110	0.040	**0.008**	0.240	0.041	**0.012**	0.360	0.042	0.340	0.729
	rs7412	-0.035	**0.019**	0.285	-0.032	**0.044**	0.660	-0.058	0.188	0.627

*P*_BH_: *P*-values after multiple adjustments using the Benjamini-Hochberg method

Total: Adjust for age, gender, BMI, smoker, drinker

Men, women: Adjust for age, BMI, smoker, drinker

### SNP and CVD

In Table 3, linear regression results revealed that variants rs3741297, rs629301, and rs2738464 were positively correlated with FRS (β = 0.031, P = 6 × 10^− 5^; β = 0.026, P = 0.001; β = 0.019, P = 0.016), whereas variants rs7780562 and rs13306194 were negatively correlated with FRS (β=-0.018, P = 0.019; β=-0.018, P = 0.022). Following BH correction, rs3741297 and rs629301 still exhibited statistically significant correlations with FRS (P_BH_<0.05). In Table 4, results from logistic regression demonstrated that the rs629301 variant increased CVD risk [OR = 1.524(95%CI:1.013-2.292), *P* = 0.043], while the rs7780562 variant decreased CVD risk [OR = 0.734(95%CI:0.601–0.895), *P* = 0.002]. Among male and female subjects, rs59379014 and rs3741297 increased CVD risk, respectively [OR = 1.42(95% CI:1.002–2.013), *P* = 0.049; OR = 2.415(95%CI:1.145–5.094), *P* = 0.021]. Nevertheless, after BH correction, none of the associations remained statistically significant.

**Table 3:** Linear regression of enrolled SNPs and FRS in our database

SNP	total			men			women		
	β	*P*	*P*_BH_	β	*P*	*P*_BH_	β	*P*	*P*_BH_
rs3741297	0.031	**6 × 10** ^**− 5**^	**0.002**	0.017	**0.033**	0.323	0.122	**5.7 × 10** ^**− 5**^	**0.002**
rs629301	0.026	**0.001**	**0.015**	0.023	**0.003**	0.090	0.049	0.109	0.314
rs2738464	0.019	**0.016**	0.132	0.013	0.092	0.394	0.062	**0.043**	0.288
rs7780562	-0.018	**0.019**	0.132	-0.008	0.317	0.732	-0.081	**0.010**	0.150
rs13306194	-0.018	**0.022**	0.132	-0.011	0.159	0.530	-0.061	**0.048**	0.288
rs12162136	-0.014	0.061	0.305	-0.008	0.301	0.732	-0.054	0.074	0.314
rs7412	-0.013	0.100	0.376	-0.016	**0.043**	0.323	0.004	0.895	0.977
rs2539981	0.012	0.116	0.376	0.015	0.062	0.370	-0.013	0.680	0.908
rs3093679	0.012	0.131	0.376	0.012	0.144	0.530	0.011	0.726	0.908
rs7703282	0.012	0.132	0.376	0.005	0.560	0.840	0.064	0.039	0.288
rs62074055	0.012	0.138	0.376	0.014	0.074	0.370	-0.001	0.977	0.977
rs2954027	-0.01	0.188	0.470	-0.007	0.385	0.765	-0.04	0.195	0.450
rs76898656	-0.01	0.220	0.508	-0.004	0.609	0.870	-0.051	0.096	0.314
rs59379014	0.009	0.263	0.564	0.018	**0.024**	0.323	-0.04	0.221	0.474
rs112784971	-0.008	0.288	0.576	-0.003	0.702	0.916	-0.045	0.145	0.363
rs6129772	-0.007	0.352	0.660	-0.006	0.456	0.765	-0.011	0.712	0.908
rs553427	0.006	0.413	0.729	0.01	0.202	0.606	-0.034	0.280	0.521
rs1501908	0.005	0.483	0.782	0	0.963	0.969	0.048	0.115	0.314
rs7140110	-0.005	0.495	0.782	-0.006	0.438	0.765	0.008	0.798	0.958
rs2642438	0.005	0.551	0.813	-0.002	0.833	0.936	0.055	0.074	0.314
rs17145738	-0.004	0.569	0.813	-0.009	0.256	0.698	0.032	0.295	0.521
rs11066015	-0.003	0.652	0.860	-0.007	0.400	0.765	0.015	0.617	0.881
rs11601507	0.003	0.663	0.860	-0.003	0.701	0.916	0.051	0.092	0.314
rs6493583	-0.003	0.688	0.860	0	0.957	0.969	-0.029	0.368	0.613
rs9376090	0.002	0.747	0.869	-0.002	0.804	0.936	0.033	0.275	0.521
rs12229026	-0.002	0.759	0.869	-0.005	0.512	0.808	0.022	0.469	0.741
rs10987829	0.002	0.782	0.869	0.006	0.459	0.765	-0.016	0.607	0.881
rs2419607	0.001	0.859	0.920	0.002	0.770	0.936	0.002	0.942	0.977
rs1883025	0	0.957	0.967	0	0.969	0.969	0.003	0.921	0.977
rs41280378	0	0.967	0.967	-0.002	0.842	0.936	0.002	0.961	0.977

*P*_BH_: *P*-values after multiple adjustments using the Benjamini-Hochberg method

Total: Adjust for age, gender, BMI, smoker, drinker

Men, women: Adjust for age, BMI, smoker, drinker

**Table 4:** Associations between SNPs with high 10-year CVD risk

SNP	OR	95%CI	*P*	*P*_BH_
total				
rs7780562	0.734	0.601–0.895	**0.002**	0.060
rs629301	1.524	1.013–2.292	**0.043**	0.473
rs3741297	1.225	0.866–1.731	0.251	0.798
rs59379014	1.196	0.873–1.638	0.266	0.798
men				
rs7780562	0.709	0.567–0.886	**0.003**	0.090
rs629301	1.48	0.954–2.298	**0.080**	0.600
rs3741297	1.012	0.682–1.5	0.954	0.995
rs59379014	1.42	1.002–2.013	**0.049**	0.490
women				
rs7780562	0.855	0.544–1.343	0.496	0.720
rs629301	1.902	0.531–6.815	0.324	0.635
rs3741297	2.415	1.145–5.094	**0.021**	0.210
rs59379014	0.557	0.221–1.403	0.215	0.595

*P*_BH_: *P*-values after multiple adjustments using the Benjamini-Hochberg method

Total: Adjust for age, gender, BMI, smoker, drinker

Men, women: Adjust for age, BMI, smoker, drinker

### SNP and IR

*Linear regression analyses were conducted to assess the associations between enrolled SNPs and continuous variables including blood glucose, insulin, and HOMA-IR, with corresponding results presented in* Tables S11-S13. *None of the associations retained statistical significance following BH correction.* In Table 5, the association between SNPs and IR among participants was examined through logistic regression analysis. The rs629301, rs10987829, rs3741297, and rs59379014 variants increased IR risk [OR = 1.253(95%CI:1.021–1.537), *P* = 0.031; OR = 1.188(95%CI:1.048–1.348), *P* = 0.007; OR = 1.222(95%CI:1.002–1.492), *P* = 0.048; OR = 1.273(95%CI:1.069–1.517), *P* = 0.007; respectively]. After BH correction, rs3741297 significantly increased IR risk among female participants [OR = 3.475 (95% CI: 1.653–7.307), P_BH_=0.03].

**Table 5:** Associations between SNPs with insulin resistance

SNP	OR	95%CI	*P*	*P*_BH_
total				
rs59379014	1.273	1.069–1.517	**0.007**	0.210
rs10987829	1.188	1.048–1.348	**0.007**	0.105
rs629301	1.253	1.021–1.537	**0.031**	0.310
rs3741297	1.222	1.002–1.492	**0.048**	0.360
men				
rs59379014	1.316	1.091–1.588	**0.004**	0.120
rs10987829	1.183	1.036–1.352	**0.013**	0.195
rs629301	1.253	1.009–1.555	**0.041**	0.410
rs3741297	1.094	0.886–1.35	0.403	0.861
women				
rs59379014	1.022	0.62–1.685	0.933	0.965
rs10987829	1.229	0.827–1.828	0.308	0.833
rs629301	1.285	0.68–2.428	0.439	0.851
rs3741297	3.475	1.653–7.307	**0.001**	**0.030**

*P*_BH_: *P*-values after multiple adjustments using the Benjamini-Hochberg method

Total: Adjust for age, gender, BMI, smoker, drinker

Men, women: Adjust for age, BMI, smoker, drinker

### SNP and MS

In Table 6, the association between SNPs and MS among participants was performed employing logistic regression analysis. In total subjects, rs1501908 and rs3741297 variants increased MS risk (OR = 1.14(95%CI: 1.024–1.271), *P* = 0.017; OR = 1.159(95% CI:1.284–1.969), *P* = 2.1 × 10^− 5^, respectively). Conversely, rs2642438, rs17145738, and rs2954027 reduced MS risk [OR = 0.844(95% CI:0.739–0.963), *P* = 0.012; OR = 0.848(95% CI:0.726–0.99), *P* = 0.036; OR = 0.899, 95% CI:0.816–0.992], *P* = 0.033, respectively]. For male subjects, rs41280378 increased MS risk [OR = 1.153(95%CI:1.026–1.295), *P* = 0.017], while rs7140110 decreased MS risk [OR = 0.869(95%CI:0.766–0.986), *P* = 0.029]. Following BH correction, rs3741297 increased MS risk [OR = 1.67(95%CI:1.369–2.038), P_BH_=1.32 × 10^− 5^], whereas rs2642438 decreased MS risk among male subjects [OR = 0.81(95%CI:0.703–0.933), P_BH_=0.045].

**Table 6:** Associations between SNPs with metabolic syndrome

SNP	OR	95%CI	*P*	*P*_BH_
total				
rs3741297	1.67	1.369–2.038	**4.4 × 10** ^**− 7**^	**1.32 × 10** ^**− 5**^
rs2642438	0.844	0.739–0.963	**0.012**	0.180
rs1501908	1.14	1.024–1.271	**0.017**	0.170
rs2954027	0.899	0.816–0.992	**0.033**	0.248
rs17145738	0.848	0.726–0.99	**0.036**	0.216
rs7140110	0.889	0.791–1	0.050	0.250
rs41280378	1.109	0.995–1.237	0.062	0.266
men				
rs3741297	1.59	1.284–1.969	**2.1 × 10** ^**− 5**^	**6.3 × 10** ^**− 4**^
rs2642438	0.81	0.703–0.933	**0.003**	**0.045**
rs1501908	1.125	1.003–1.262	**0.045**	0.225
rs2954027	0.904	0.815–1.004	0.059	0.253
rs17145738	0.831	0.705–0.979	**0.027**	0.174
rs7140110	0.869	0.766–0.986	**0.029**	0.174
rs41280378	1.153	1.026–1.295	**0.017**	0.170
women				
rs3741297	2.347	1.339–4.115	**0.003**	0.090
rs2642438	1.147	0.772–1.703	0.497	0.766
rs1501908	1.3	0.941–1.796	0.112	0.480
rs2954027	0.861	0.649–1.14	0.295	0.702
rs17145738	1.007	0.618–1.642	0.977	0.977
rs7140110	1.008	0.731–1.389	0.961	0.977
rs41280378	0.863	0.627–1.187	0.366	0.720

*P*_BH_: *P*-values after multiple adjustments using the Benjamini-Hochberg method

Total: Adjust for age, gender, BMI, smoker, drinker

Men, women: Adjust for age, BMI, smoker, drinker

## Discussion

This study delineated the relationship between LDL-C genetic variants and CVD, IR, as well as MS in an OSA population. Multiple LDL-C SNPs were associated with OSA related parameters, CVD, IR or MS in our study. Following adjustment utilizing the BH method, rs3741297 emerged as a significant contributor to increased susceptibility to CVD, IR, and MS among subjects. The findings suggest the potential presence of a shared genetic background among these conditions.

OSA, CVD, IR and MS are complex, polygenic conditions with a shared pathophysiological foundation, including ectopic lipid accumulation, adipokine dysregulation, oxidative stress, systemic inflammation, and disturbances in intestinal flora [28–32]. Our study highlighted the significant role of genetic factors in dyslipidemia, contributing potentially to the development of these diseases. The rs629301 variant, a potent SNP heightening LDL-C levels, is thought to be independently associated with CVD [33], and it increased subjects’ susceptibility to CVD and IR in our study. The rs13306194 variant, linked to diminished LDL-C levels [34], exhibited a negative correlation with FRS in our subjects. Moreover, carriers of the rs1501908 variant, featuring elevated LDL-C levels [35], showed a heightened risk of MS. In addition, the rs2738464-C allele may affect the affinity of the LDL-C receptor, thereby impairing its regulation of cholesterol homeostasis [36], and this variant was positively associated with FRS in our cohort. Although the rs3741297 and rs17145738 variants were initially enrolled as SNPs influencing LDL-C levels, our study revealed that they exhibited a stronger effect on HDL-C levels, aligning with previous studies [37, 38]. The rs3741297 variant in Zinc-finger protein1 (ZPR1) gene significantly reduces HDL-C levels [37], and this variant increased the susceptibility to CVD and IR in our female subjects, which needs to be validated in a larger female cohort. The rs17145738-T allele correlated with higher HDL-C levels in Han Chinese males [38], which indirectly supports our finding that it displayed a protective effect against MS in male subjects. The rs7412 variant has demonstrated a significant effect on lipid profile and cognitive performance in aging Chinese population [39], and it exhibited negative correlations with FRS and MAI in our results. Nevertheless, within the aforementioned SNPs, solely rs629301 and rs3741297 passed multiple testing correction, indicating potential false positive associations with the remaining SNPs and the disease phenotype, warranting cautious interpretation. The intrinsic nature of multiple testing correction mandates increased stringency with test number escalation. With up to 30 corrections in this study, smaller effect SNP associations with the disease phenotype may be obscured, leading to false negatives. Additionally, factors such as data quality, phenotypic complexity, and gene-environment interactions may further influence results. To elucidate unresolved SNP variations, a more thorough experimental approach is required. This could involve enlarging sample sizes, employing alternative analytical paradigms, and exploring gene-environment interactions.

Apart from their impact on lipid profiles, certain SNPs are correlated with the modulation of pivotal genes, thereby influencing the pathogenesis of diseases. For instance, the rs2539981 variant in EH Domain-Binding Protein 1 (EHBP1) gene exhibited increased expression of the orthodenticle homeobox 1 (Otx1), implicated in cerebral cortex development [40], and this variant reduced MAI in our results. The rs7140110 variant inhibited expression of the Growth arrest-specific protein 6 (GAS6), a protein reported to be associated with IR and systemic inflammation [41], and was protective against HOMA-IR and MS in our male subjects. The rs7780562 variant reduced the expression of the Sorting Nexin 10 (SNX10), a protein associated with diet-induced atherosclerosis [42], and may reduce CVD risk in our subjects through this pathway. Notably, the rs7780562 appears to correlated with hypoxia, displaying association with heightened respiratory rate and diminished exertional lung capacity within the chronic obstructive pulmonary disease (COPD) population [43], which was linked to increased AHI and ODI within our OSA population. The rs2642438 variant in the Mitochondrial Amidoxime Reducing Component 1 (MARC1) gene exhibited varied metabolic effects across ethnicities. In European populations, the rs2642438-A allele has correlated with protection against fatty liver [44]. However, this association was not observed regarding the risk of non-alcoholic fatty liver disease (NAFLD) in Korean populations [45]. In our study involving the Chinese population, the rs2642438-G allele exhibited a protective effect against MS. Our inquiry observed an increased predisposition to CVD and IR linked to the rs59379014 variant in the ST3 beta-galactoside alpha-2,3-sialyltransferase 4 (ST3GAL4) gene. Additionally, the rs2954027 variant in the Tribbles Pseudokinase 1 (TRIB1) gene exhibited correlations with diminished ODI and a decreased risk of MS in subjects. Given the close associations between specific genes related to LDL-C and the diseases, subsequent research focusing on genes rather than SNPs may reduce the number of multiple tests, thereby enhancing statistical power.

### Strengths and limitations

Our investigation into genetic variation within LDL-C enriched genetic links to OSA, CVD, IR, and MS through robust analysis with large sample sizes, sex-stratified evaluations, and ethnically appropriate SNPs. However, there are some limitations in this study that warrant consideration. Primarily, the cross-sectional design prevents establishing causality. Secondly, the extensive array of SNPs mandates rigorous BH correction, leading to the attenuation of significance in SNP-disease associations following adjustment. Subsequent research focusing on genes or metabolic pathways holds promise for enhancing statistical efficacy. Furthermore, the limited female sample restricts the broader applicability of our findings to the female population and impedes a thorough exploration of gender-specific effects on cardiovascular metabolism. Lastly, despite adjustments for smoking and alcohol consumption as confounding factors throughout our statistical analyses, other lifestyle factors influencing cardiovascular and metabolic outcomes, such as dietary habits and physical activity, were not comprehensively investigated. It is hoped that more detailed and comprehensive studies will address these limitations and provide new explanations and sufficient evidence for the intricate genetic roles among cardiovascular metabolic diseases.

## Conclusion

Our research has identified a distinct association between specific LDL-C SNPs and an elevated susceptibility to OSA, CVD, IR, and MS. Noteworthy, a shared genetic basis appears to underlie the pathogenesis of these diseases. The identified variants have potential applications in managing cardiovascular and metabolic disorders, including screening high-risk population and developing targeted therapies.

### Electronic supplementary material

Below is the link to the electronic supplementary material.

Supplementary Material 1

## Data Availability

No datasets were generated or analysed during the current study.
