# Supplementary material for: Genetic variations of low-density lipoprotein cholesterol on metabolic disorders in obstructive sleep apnea
Source: Nutr Metab (Lond). 2024 Jun 10;21:31. doi: 10.1186/s12986-024-00805-z (PMC11163771; doi:10.1186/s12986-024-00805-z)
Supplement: Supplementary file 1 — Supplementary Material 1 [file 12986_2024_805_MOESM1_ESM.docx]

**Figure S1 Process for inclusion and exclusion of subjects**


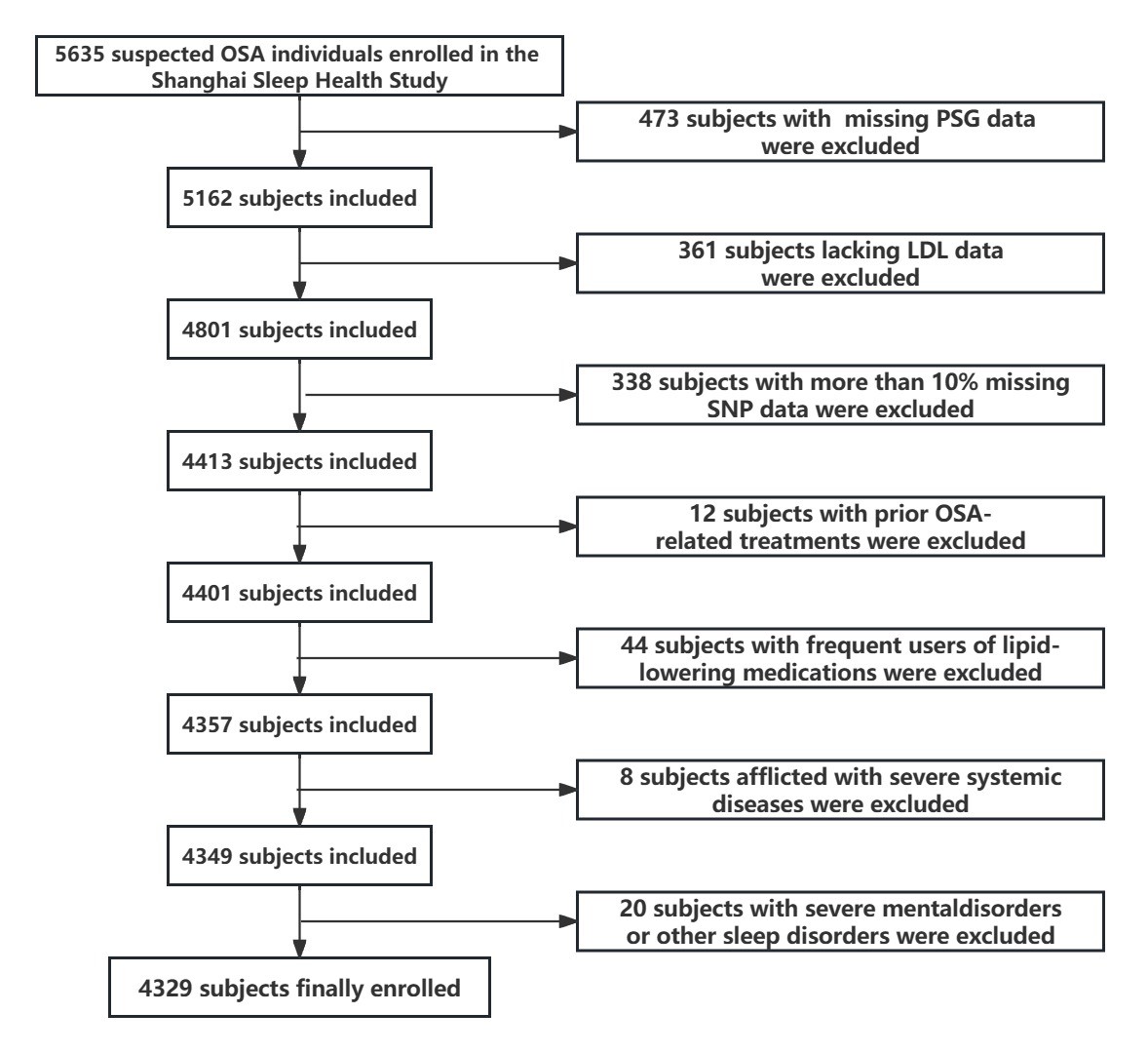


**OSA，obstructive sleep apnea; PSG, polysomnography; LDL-C, low-density lipoprotein cholesterol.**

**Figure S2 Selection and quality control process of SNPs**


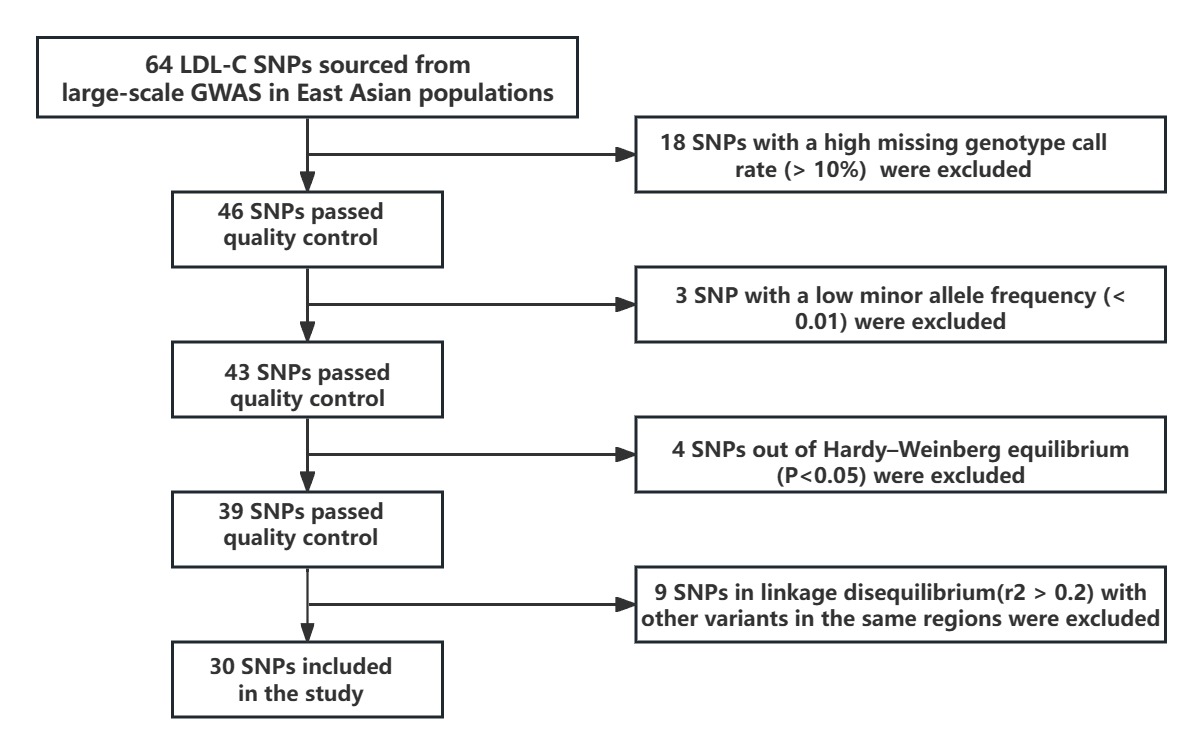


**SNP, single nucleotide polymorphism; LDL-C, low-density lipoprotein** **cholesterol.**

**Table S1 basic Information on enrolled SNPs**

| SNP | Gene | Risk allele | Major  allele | Minor  allele | MAF | Ch | Position |
| --- | --- | --- | --- | --- | --- | --- | --- |
| rs553427 | LINC01132 | T | T | C | 0.241 | 1 | 234852760 |
| rs629301 | CELSR2 | T | T | G | 0.06 | 1 | 109818306 |
| rs2642438 | MTARC1 | G | G | A | 0.152 | 1 | 220970028 |
| rs2539981 | EHBP1 | C | C | T | 0.336 | 2 | 63104904 |
| rs13306194 | APOB | A | G | A | 0.113 | 2 | 21252534 |
| rs1501908 | HAVCR1 | C | C | G | 0.26 | 5 | 156398169 |
| rs7703282 | POLK | C | C | A | 0.466 | 5 | 74906963 |
| rs9376090 | HBS1L | C | T | C | 0.285 | 6 | 135411228 |
| rs17145738 | TBL2 | T | C | T | 0.102 | 7 | 72982874 |
| rs7780562 | MIR148A | C | C | A | 0.306 | 7 | 26022414 |
| rs112784971 | CYP7A1 | T | C | T | 0.237 | 8 | 59406490 |
| rs2954027 | TRIB1 | A | A | T | 0.415 | 8 | 126485294 |
| rs10987829 | EEIG1 | G | A | G | 0.19 | 9 | 130776743 |
| rs1883025 | ABCA1 | T | C | T | 0.213 | 9 | 107664301 |
| rs41280378 | OIT3 | G | T | G | 0.266 | 10 | 74692646 |
| rs2419607 | GPAM | G | T | G | 0.414 | 10 | 113978499 |
| rs3741297 | ZPR1 | T | C | T | 0.065 | 11 | 116657667 |
| rs59379014 | ST3GAL4 | T | C | T | 0.098 | 11 | 126228000 |
| rs11601507 | TRIM5 | A | C | A | 0.101 | 11 | 5701074 |
| rs11066015 | ACAD10 | A | G | A | 0.204 | 12 | 112168009 |
| rs12229026 | NR1H4, | C | T | C | 0.304 | 12 | 100834253 |
| rs7140110 | GAS6-AS1 | C | T | C | 0.227 | 13 | 114544024 |
| rs76898656 | ALDH1A2 | A | G | A | 0.338 | 15 | 58691202 |
| rs6493583 | ONECUT1 | G | C | G | 0.168 | 15 | 53096084 |
| rs3093679 | TNFAIP1 | A | G | A | 0.218 | 17 | 26664215 |
| rs12162136 | ABCA6 | A | A | G | 0.379 | 17 | 67082266 |
| rs62074055 | TBKBP1 | C | G | C | 0.339 | 17 | 45771933 |
| rs7412 | APOE | T | C | T | 0.077 | 19 | 45412079 |
| rs2738464 | SPC24 | C | C | G | 0.293 | 19 | 11242307 |
| rs6129772 | ZHX3 | T | T | C | 0.238 | 20 | 39845070 |

**MAF, minor allele frequency; Ch, chromosome.**

**Information of minor allele frequency and position was obtained from our database.**

**Table S2 Linear regression of enrolled SNPs and LDL-C levels in our database**

| SNP | total |  |  | men |  |  | women |  |  |
| --- | --- | --- | --- | --- | --- | --- | --- | --- | --- |
|  | β | P | P_BH_ | β | P | P_BH_ | β | P | P_BH_ |
| rs7412 | -0.217 | **2×10^-45^** | **6×10^-44^** | -0.204 | **4×10^-36^** | **1×10^-34^** | -0.298 | **2×10^-11^** | **6×10^-10^** |
| rs629301 | 0.1 | **4×10^-11^** | **7×10^-10^** | 0.092 | **1×10^-8^** | **2×10^-7^** | 0.153 | **0.001** | **0.015** |
| rs2738464 | 0.072 | **3×10^-6^** | **3×10^-5^** | 0.069 | **2×10^-5^** | **2×10^-4^** | 0.09 | **0.044** | 0.220 |
| rs3741297 | -0.067 | **9×10^-6^** | **7×10^-5^** | -0.072 | **1×10^-5^** | **1×10^-4^** | -0.028 | 0.538 | 0.807 |
| rs7703282 | 0.045 | **0.004** | **0.024** | 0.038 | **0.020** | 0.086 | 0.095 | **0.037** | 0.220 |
| rs13306194 | -0.041 | **0.009** | **0.045** | -0.053 | **0.001** | **0.006** | 0.042 | 0.357 | 0.703 |
| rs7780562 | -0.037 | **0.016** | 0.069 | -0.034 | **0.037** | 0.139 | -0.057 | 0.212 | 0.578 |
| rs6129772 | 0.032 | **0.033** | 0.124 | 0.027 | 0.096 | 0.255 | 0.064 | 0.15 | 0.500 |
| rs11601507 | 0.029 | 0.058 | 0.193 | 0.023 | 0.161 | 0.308 | 0.071 | 0.111 | 0.416 |
| rs59379014 | 0.028 | 0.077 | 0.225 | 0.015 | 0.392 | 0.535 | 0.117 | **0.013** | 0.130 |
| rs41280378 | -0.025 | 0.100 | 0.225 | -0.04 | **0.015** | 0.075 | 0.086 | 0.055 | 0.236 |
| rs9376090 | -0.025 | 0.107 | 0.225 | -0.03 | 0.066 | 0.220 | 0.02 | 0.657 | 0.821 |
| rs1501908 | 0.024 | 0.111 | 0.225 | 0.023 | 0.164 | 0.270 | 0.036 | 0.413 | 0.832 |
| rs17145738 | 0.024 | 0.111 | 0.225 | 0.025 | 0.117 | 0.308 | 0.018 | 0.693 | 0.729 |
| rs2954027 | -0.024 | 0.116 | 0.225 | -0.027 | 0.094 | 0.255 | 0.008 | 0.858 | 0.888 |
| rs3093679 | -0.024 | 0.120 | 0.225 | -0.021 | 0.204 | 0.353 | -0.04 | 0.375 | 0.703 |
| rs112784971 | -0.023 | 0.133 | 0.235 | -0.023 | 0.148 | 0.308 | -0.015 | 0.729 | 0.841 |
| rs6493583 | -0.023 | 0.144 | 0.240 | -0.021 | 0.212 | 0.353 | -0.035 | 0.464 | 0.733 |
| rs2539981 | 0.021 | 0.171 | 0.270 | 0.027 | 0.102 | 0.255 | -0.014 | 0.757 | 0.841 |
| rs2642438 | 0.015 | 0.316 | 0.474 | 0.15 | 0.354 | 0.506 | 0.008 | 0.855 | 0.888 |
| rs11066015 | 0.014 | 0.369 | 0.527 | 0.019 | 0.244 | 0.385 | -0.022 | 0.619 | 0.810 |
| rs12162136 | -0.013 | 0.401 | 0.547 | -0.008 | 0.639 | 0.696 | -0.05 | 0.262 | 0.605 |
| rs553427 | 0.011 | 0.456 | 0.584 | 0.015 | 0.346 | 0.506 | 0 | 0.997 | 0.997 |
| rs76898656 | -0.011 | 0.467 | 0.584 | -0.009 | 0.594 | 0.696 | -0.023 | 0.604 | 0.810 |
| rs10987829 | 0.01 | 0.493 | 0.592 | 0.008 | 0.616 | 0.696 | 0.022 | 0.621 | 0.810 |
| rs12229026 | 0.006 | 0.699 | 0.807 | -0.007 | 0.650 | 0.696 | 0.096 | 0.034 | 0.220 |
| rs7140110 | 0.005 | 0.735 | 0.817 | 0.012 | 0.489 | 0.611 | -0.047 | 0.305 | 0.654 |
| rs2419607 | -0.003 | 0.830 | 0.889 | -0.013 | 0.435 | 0.567 | 0.059 | 0.195 | 0.578 |
| rs1883025 | -0.002 | 0.907 | 0.938 | 0.005 | 0.770 | 0.779 | -0.053 | 0.243 | 0.605 |
| rs62074055 | 0 | 0.979 | 0.979 | 0.005 | 0.779 | 0.779 | -0.034 | 0.457 | 0.733 |

**P_BH_: P-values after multiple adjustments using the Benjamini-Hochberg method.**

**Total: Adjust for age, gender, BMI, smoker, drinker.**

**Men, women: Adjust for age, BMI, smoker, drinker.**

**Table S3 Linear regression of enrolled SNPs and HDL-C levels in our database**

| SNP | total |  |  | men |  |  | women |  |  |
| --- | --- | --- | --- | --- | --- | --- | --- | --- | --- |
|  | β | P | P_BH_ | β | P | P_BH_ | β | P | P_BH_ |
| rs3741297 | -0.128 | **1×10^-18^** | **3×10^-17^** | -0.121 | **3×10^-14^** | **9×10^-13^** | -0.188 | **1×10^-5^** | **3×10^-4^** |
| rs11066015 | -0.043 | **0.003** | **0.045** | -0.047 | **0.003** | **0.030** | -0.029 | 0.501 | 0.840 |
| rs76898656 | -0.041 | **0.005** | 0.050 | -0.038 | **0.019** | 0.114 | -0.071 | 0.099 | 0.743 |
| rs1883025 | -0.037 | **0.011** | 0.066 | -0.048 | **0.003** | 0.030 | 0.013 | 0.756 | 0.935 |
| rs2419607 | -0.038 | **0.011** | 0.083 | -0.047 | **0.004** | 0.030 | 0.008 | 0.860 | 0.872 |
| rs41280378 | -0.03 | **0.041** | 0.189 | -0.034 | **0.036** | 0.135 | -0.014 | 0.751 | 0.872 |
| rs12162136 | 0.03 | **0.044** | 0.189 | 0.029 | 0.072 | 0.216 | 0.045 | 0.292 | 0.840 |
| rs6129772 | 0.027 | 0.064 | 0.240 | 0.027 | 0.089 | 0.243 | 0.028 | 0.507 | 0.840 |
| rs17145738 | 0.026 | 0.075 | 0.250 | 0.036 | **0.023** | 0.115 | -0.029 | 0.493 | 0.840 |
| rs2539981 | -0.024 | 0.106 | 0.318 | -0.017 | 0.296 | 0.531 | -0.061 | 0.155 | 0.840 |
| rs2642438 | 0.023 | 0.125 | 0.330 | 0.033 | 0.043 | 0.143 | -0.045 | 0.297 | 0.840 |
| rs7412 | 0.022 | 0.132 | 0.330 | 0.019 | 0.243 | 0.486 | 0.049 | 0.257 | 0.840 |
| rs112784971 | 0.021 | 0.160 | 0.356 | 0.021 | 0.183 | 0.422 | 0.028 | 0.506 | 0.840 |
| rs9376090 | 0.02 | 0.166 | 0.356 | 0.02 | 0.221 | 0.474 | 0.023 | 0.588 | 0.840 |
| rs10987829 | 0.017 | 0.246 | 0.476 | 0.017 | 0.301 | 0.531 | 0.028 | 0.514 | 0.840 |
| rs2738464 | -0.017 | 0.254 | 0.476 | -0.012 | 0.451 | 0.712 | -0.045 | 0.294 | 0.840 |
| rs13306194 | 0.015 | 0.313 | 0.552 | 0.011 | 0.519 | 0.720 | 0.041 | 0.352 | 0.840 |
| rs553427 | 0.012 | 0.397 | 0.662 | 0.016 | 0.320 | 0.533 | -0.001 | 0.974 | 0.974 |
| rs3093679 | -0.012 | 0.427 | 0.674 | -0.006 | 0.711 | 0.927 | -0.044 | 0.317 | 0.840 |
| rs7703282 | -0.01 | 0.488 | 0.717 | -0.01 | 0.528 | 0.720 | -0.014 | 0.754 | 0.872 |
| rs12229026 | 0.01 | 0.506 | 0.717 | 0.011 | 0.483 | 0.720 | -0.004 | 0.931 | 0.963 |
| rs59379014 | -0.01 | 0.526 | 0.717 | -0.036 | 0.032 | 0.135 | 0.151 | 0.001 | 0.015 |
| rs7140110 | 0.007 | 0.638 | 0.832 | 0.023 | 0.171 | 0.422 | -0.091 | 0.038 | 0.380 |
| rs6493583 | 0.005 | 0.726 | 0.893 | 0.001 | 0.972 | 0.991 | 0.033 | 0.473 | 0.840 |
| rs7780562 | 0.005 | 0.744 | 0.893 | 0 | 0.981 | 0.991 | 0.044 | 0.318 | 0.840 |
| rs629301 | -0.004 | 0.796 | 0.897 | -0.002 | 0.894 | 0.991 | -0.017 | 0.690 | 0.872 |
| rs62074055 | 0.004 | 0.807 | 0.897 | 0.002 | 0.883 | 0.991 | 0.024 | 0.582 | 0.840 |
| rs11601507 | -0.003 | 0.849 | 0.898 | 0 | 0.991 | 0.991 | -0.017 | 0.692 | 0.872 |
| rs2954027 | 0.002 | 0.868 | 0.898 | -0.001 | 0.931 | 0.991 | 0.026 | 0.545 | 0.840 |
| rs1501908 | -0.001 | 0.953 | 0.953 | 0 | 0.979 | 0.991 | -0.007 | 0.873 | 0.935 |

**P_BH_: P-values after multiple adjustments using the Benjamini-Hochberg method.**

**Total: Adjust for age, gender, BMI, smoker, drinker.**

**Men, women: Adjust for age, BMI, smoker, drinker.**

**Table S4 Linear regression of enrolled SNPs and TC levels in our database**

| SNP | total |  |  | men |  |  | women |  |  |
| --- | --- | --- | --- | --- | --- | --- | --- | --- | --- |
|  | β | P | P_BH_ | β | P | P_BH_ | β | P | P_BH_ |
| rs629301 | 0.112 | **1×10^-13^** | **3×10^-12^** | 0.109 | **2×10^-11^** | **6×10^-10^** | 0.129 | **0.004** | **0.060** |
| rs7412 | -0.082 | **1×10^-7^** | **2×10^-6^** | -0.068 | **4×10^-5^** | **4×10^-4^** | -0.181 | **5×10^-5^** | **0.002** |
| rs2954027 | -0.067 | **9×10^-6^** | **9×10^-5^** | -0.067 | **3×10^-5^** | **5×10^-4^** | -0.057 | 0.201 | 0.573 |
| rs2738464 | 0.059 | **1×10^-4^** | **0.001** | 0.055 | **0.001** | **0.008** | 0.089 | **0.043** | 0.215 |
| rs76898656 | -0.049 | **0.001** | **0.006** | -0.048 | **0.003** | **0.015** | -0.05 | 0.257 | 0.616 |
| rs13306194 | -0.042 | **0.007** | **0.023** | -0.049 | **0.003** | **0.015** | 0.002 | 0.956 | 0.956 |
| rs7703282 | 0.042 | **0.007** | **0.023** | 0.042 | **0.012** | 0.051 | 0.038 | 0.399 | 0.665 |
| rs3741297 | 0.041 | **0.007** | **0.023** | 0.036 | **0.028** | 0.100 | 0.103 | **0.019** | 0.143 |
| rs59379014 | 0.043 | **0.007** | **0.023** | 0.029 | 0.083 | 0.178 | 0.125 | **0.007** | 0.070 |
| rs7780562 | -0.037 | **0.016** | **0.048** | -0.03 | 0.073 | 0.178 | -0.091 | **0.041** | 0.215 |
| rs112784971 | -0.036 | **0.018** | **0.049** | -0.035 | **0.030** | 0.100 | -0.035 | 0.431 | 0.681 |
| rs9376090 | -0.03 | **0.049** | 0.123 | -0.032 | **0.046** | 0.125 | -0.009 | 0.839 | 0.948 |
| rs7140110 | 0.028 | 0.076 | 0.162 | 0.035 | **0.034** | 0.102 | -0.039 | 0.390 | 0.665 |
| rs6493583 | -0.028 | 0.08 | 0.162 | -0.025 | 0.145 | 0.272 | -0.048 | 0.308 | 0.616 |
| rs1501908 | 0.027 | 0.081 | 0.162 | 0.022 | 0.166 | 0.293 | 0.056 | 0.206 | 0.573 |
| rs1883025 | -0.025 | 0.104 | 0.195 | -0.021 | 0.186 | 0.310 | -0.056 | 0.210 | 0.573 |
| rs2539981 | 0.024 | 0.120 | 0.212 | 0.028 | 0.080 | 0.178 | 0.005 | 0.916 | 0.948 |
| rs11601507 | 0.017 | 0.256 | 0.411 | 0.011 | 0.502 | 0.602 | 0.06 | 0.172 | 0.573 |
| rs3093679 | -0.017 | 0.260 | 0.411 | -0.012 | 0.468 | 0.602 | -0.046 | 0.304 | 0.616 |
| rs2642438 | 0.017 | 0.278 | 0.417 | 0.014 | 0.381 | 0.520 | 0.018 | 0.680 | 0.850 |
| rs11066015 | -0.016 | 0.308 | 0.440 | -0.017 | 0.295 | 0.421 | -0.005 | 0.906 | 0.948 |
| rs41280378 | -0.015 | 0.324 | 0.442 | -0.026 | 0.110 | 0.220 | 0.073 | 0.103 | 0.441 |
| rs12162136 | -0.013 | 0.392 | 0.504 | -0.011 | 0.498 | 0.602 | -0.026 | 0.559 | 0.762 |
| rs10987829 | 0.013 | 0.403 | 0.504 | 0.019 | 0.250 | 0.390 | -0.041 | 0.356 | 0.665 |
| rs553427 | 0.01 | 0.523 | 0.623 | 0.018 | 0.26 | 0.390 | -0.027 | 0.546 | 0.762 |
| rs12229026 | 0.009 | 0.540 | 0.623 | 0.002 | 0.881 | 0.911 | 0.046 | 0.299 | 0.616 |
| rs17145738 | -0.008 | 0.581 | 0.646 | -0.008 | 0.634 | 0.704 | -0.006 | 0.884 | 0.948 |
| rs6129772 | 0.007 | 0.640 | 0.677 | 0.003 | 0.861 | 0.911 | 0.026 | 0.55 | 0.762 |
| rs62074055 | 0.007 | 0.654 | 0.677 | 0.01 | 0.537 | 0.620 | -0.009 | 0.841 | 0.948 |
| rs2419607 | 0.004 | 0.797 | 0.797 | -0.001 | 0.974 | 0.974 | 0.024 | 0.590 | 0.770 |

**P_BH_: P-values after multiple adjustments using the Benjamini-Hochberg method.**

**Total: Adjust for age, gender, BMI, smoker, drinker.**

**Men, women: Adjust for age, BMI, smoker, drinker.**

**Table S5 Linear regression of enrolled SNPs and TG levels in our database**

| SNP | total |  |  | men |  |  | women |  |  |
| --- | --- | --- | --- | --- | --- | --- | --- | --- | --- |
|  | β | P | P_BH_ | β | P | P_BH_ | β | P | P_BH_ |
| rs3741297 | 0.195 | **2×10^-38^** | **6×10^-37^** | 0.191 | **1×10^-32^** | **3×10^-31^** | 0.239 | **6×10^-8^** | **2×10^-6^** |
| rs7412 | 0.079 | **3×10^-7^** | **5×10^-6^** | 0.091 | **3×10^-8^** | **5×10^-7^** | 0.001 | 0.979 | 0.990 |
| rs2954027 | -0.066 | **1×10^-5^** | **1×10^-4^** | -0.067 | **3×10^-5^** | **3×10^-4^** | -0.053 | 0.238 | 0.621 |
| rs17145738 | -0.05 | **0.001** | **0.006** | -0.053 | **0.001** | **0.008** | -0.021 | 0.631 | 0.947 |
| rs112784971 | -0.049 | **0.001** | **0.006** | -0.048 | **0.003** | **0.018** | -0.057 | 0.199 | 0.621 |
| rs6129772 | -0.037 | **0.016** | 0.080 | -0.037 | **0.024** | 0.120 | -0.046 | 0.301 | 0.621 |
| rs9376090 | -0.03 | 0.051 | 0.219 | -0.027 | 0.096 | 0.262 | -0.052 | 0.246 | 0.621 |
| rs2419607 | 0.029 | 0.063 | 0.236 | 0.036 | 0.031 | 0.120 | -0.035 | 0.444 | 0.718 |
| rs629301 | 0.026 | 0.082 | 0.273 | 0.035 | **0.032** | 0.120 | -0.045 | 0.317 | 0.621 |
| rs41280378 | 0.026 | 0.093 | 0.279 | 0.03 | 0.063 | 0.210 | -0.007 | 0.873 | 0.970 |
| rs7140110 | 0.023 | 0.136 | 0.371 | 0.019 | 0.246 | 0.492 | 0.048 | 0.289 | 0.621 |
| rs76898656 | -0.02 | 0.184 | 0.433 | -0.023 | 0.148 | 0.317 | 0.011 | 0.812 | 0.970 |
| rs1883025 | -0.02 | 0.199 | 0.433 | -0.016 | 0.312 | 0.548 | -0.042 | 0.345 | 0.621 |
| rs11066015 | -0.019 | 0.202 | 0.433 | -0.028 | 0.088 | 0.262 | 0.048 | 0.276 | 0.621 |
| rs13306194 | -0.018 | 0.244 | 0.458 | -0.015 | 0.350 | 0.284 | -0.042 | 0.350 | 0.621 |
| rs7703282 | 0.018 | 0.244 | 0.488 | 0.026 | 0.119 | 0.548 | -0.042 | 0.352 | 0.621 |
| rs3093679 | 0.013 | 0.410 | 0.665 | 0.015 | 0.365 | 0.548 | 0.005 | 0.909 | 0.974 |
| rs7780562 | 0.012 | 0.427 | 0.665 | 0.025 | 0.123 | 0.284 | -0.091 | 0.043 | 0.621 |
| rs59379014 | 0.013 | 0.430 | 0.665 | 0.017 | 0.329 | 0.548 | -0.035 | 0.455 | 0.718 |
| rs553427 | -0.012 | 0.443 | 0.665 | -0.006 | 0.708 | 0.817 | -0.043 | 0.340 | 0.621 |
| rs6493583 | -0.01 | 0.544 | 0.777 | -0.001 | 0.934 | 0.934 | -0.07 | 0.134 | 0.621 |
| rs11601507 | -0.007 | 0.661 | 0.821 | -0.01 | 0.541 | 0.719 | 0.017 | 0.701 | 0.956 |
| rs62074055 | 0.007 | 0.663 | 0.821 | 0.008 | 0.616 | 0.739 | -0.001 | 0.990 | 0.990 |
| rs2539981 | 0.007 | 0.667 | 0.821 | 0.01 | 0.530 | 0.719 | -0.015 | 0.739 | 0.964 |
| rs10987829 | 0.006 | 0.686 | 0.821 | 0.015 | 0.351 | 0.548 | -0.074 | 0.096 | 0.621 |
| rs12162136 | -0.005 | 0.728 | 0.821 | -0.009 | 0.591 | 0.739 | 0.019 | 0.673 | 0.956 |
| rs12229026 | -0.005 | 0.739 | 0.821 | -0.005 | 0.743 | 0.826 | -0.009 | 0.848 | 0.970 |
| rs2738464 | 0.003 | 0.850 | 0.911 | -0.004 | 0.829 | 0.858 | 0.048 | 0.283 | 0.621 |
| rs2642438 | -0.002 | 0.902 | 0.913 | -0.004 | 0.785 | 0.841 | 0.01 | 0.823 | 0.970 |
| rs1501908 | -0.002 | 0.913 | 0.913 | -0.01 | 0.551 | 0.719 | 0.068 | 0.128 | 0.960 |

**P_BH_: P-values after multiple adjustments using the Benjamini-Hochberg method.**

**Total: Adjust for age, gender, BMI, smoker, drinker.**

**Men, women: Adjust for age, BMI, smoker, drinker.**

**Table S6 Linear regression of enrolled SNPs and AHI in our database**

| SNP | total |  |  | men |  |  | women |  |  |
| --- | --- | --- | --- | --- | --- | --- | --- | --- | --- |
|  | β | P | P_BH_ | β | P | P_BH_ | β | P | P_BH_ |
| rs7780562 | 0.036 | **0.01** | 0.300 | 0.028 | 0.054 | 0.438 | 0.074 | 0.083 | 0.959 |
| rs7412 | -0.026 | 0.063 | 0.630 | -0.026 | 0.073 | 0.438 | -0.015 | 0.722 | 0.959 |
| rs76898656 | -0.025 | 0.068 | 0.630 | -0.032 | **0.024** | 0.438 | 0.032 | 0.447 | 0.959 |
| rs10987829 | -0.024 | 0.084 | 0.630 | -0.027 | 0.059 | 0.438 | -0.011 | 0.797 | 0.967 |
| rs6129772 | -0.02 | 0.150 | 0.692 | -0.028 | 0.055 | 0.438 | 0.037 | 0.386 | 0.959 |
| rs9376090 | -0.02 | 0.152 | 0.692 | -0.019 | 0.188 | 0.863 | -0.017 | 0.687 | 0.959 |
| rs41280378 | -0.018 | 0.185 | 0.692 | -0.012 | 0.400 | 0.909 | -0.049 | 0.252 | 0.959 |
| rs7703282 | -0.018 | 0.204 | 0.692 | -0.018 | 0.234 | 0.863 | -0.004 | 0.921 | 0.967 |
| rs2954027 | -0.016 | 0.258 | 0.692 | -0.012 | 0.424 | 0.909 | -0.038 | 0.372 | 0.959 |
| rs2738464 | -0.015 | 0.261 | 0.692 | -0.017 | 0.248 | 0.863 | -0.033 | 0.439 | 0.959 |
| rs1501908 | 0.015 | 0.273 | 0.692 | 0.016 | 0.259 | 0.863 | 0.02 | 0.640 | 0.959 |
| rs7140110 | 0.015 | 0.295 | 0.692 | 0.015 | 0.314 | 0.909 | 0.002 | 0.967 | 0.967 |
| rs12229026 | -0.014 | 0.300 | 0.692 | -0.001 | 0.934 | 0.966 | -0.124 | 0.004 | 0.120 |
| rs11066015 | -0.011 | 0.409 | 0.801 | -0.012 | 0.397 | 0.909 | 0.029 | 0.491 | 0.959 |
| rs11601507 | -0.011 | 0.427 | 0.801 | -0.009 | 0.553 | 0.909 | -0.033 | 0.442 | 0.967 |
| rs3741297 | -0.011 | 0.427 | 0.854 | -0.013 | 0.351 | 0.966 | 0.003 | 0.942 | 0.959 |
| rs553427 | -0.009 | 0.511 | 0.856 | -0.005 | 0.713 | 0.966 | -0.036 | 0.400 | 0.959 |
| rs629301 | -0.009 | 0.524 | 0.856 | -0.002 | 0.909 | 0.966 | -0.044 | 0.304 | 0.959 |
| rs1883025 | -0.008 | 0.542 | 0.856 | -0.002 | 0.901 | 0.966 | -0.018 | 0.679 | 0.959 |
| rs13306194 | -0.007 | 0.615 | 0.870 | -0.003 | 0.813 | 0.966 | -0.046 | 0.292 | 0.959 |
| rs6493583 | 0.007 | 0.629 | 0.870 | 0.009 | 0.558 | 0.966 | 0.015 | 0.735 | 0.959 |
| rs2642438 | -0.006 | 0.668 | 0.870 | -0.005 | 0.719 | 0.966 | -0.017 | 0.682 | 0.959 |
| rs2539981 | -0.005 | 0.705 | 0.870 | -0.005 | 0.735 | 0.966 | 0.006 | 0.881 | 0.967 |
| rs2419607 | -0.005 | 0.719 | 0.870 | -0.008 | 0.564 | 0.966 | 0.006 | 0.884 | 0.967 |
| rs59379014 | 0.005 | 0.725 | 0.870 | 0.003 | 0.829 | 0.966 | -0.017 | 0.707 | 0.959 |
| rs62074055 | -0.004 | 0.796 | 0.884 | -0.007 | 0.641 | 0.966 | 0.017 | 0.698 | 0.959 |
| rs3093679 | 0.003 | 0.810 | 0.884 | 0.001 | 0.966 | 0.966 | 0.033 | 0.449 | 0.959 |
| rs112784971 | 0.003 | 0.825 | 0.884 | -0.002 | 0.870 | 0.966 | 0.03 | 0.484 | 0.959 |
| rs12162136 | -0.002 | 0.870 | 0.895 | -0.008 | 0.584 | 0.966 | 0.009 | 0.831 | 0.967 |
| rs17145738 | 0.002 | 0.895 | 0.895 | -0.003 | 0.828 | 0.966 | 0.042 | 0.324 | 0.959 |

**P_BH_: P-values after multiple adjustments using the Benjamini-Hochberg method.**

**Total: Adjust for age, gender, BMI, smoker, drinker.**

**Men, women: Adjust for age, BMI, smoker, drinker.**

**Table S7 Linear regression of enrolled SNPs and ODI in our database**

| SNP | total |  |  | men |  |  | women |  |  |
| --- | --- | --- | --- | --- | --- | --- | --- | --- | --- |
|  | β | P | P_BH_ | β | P | P_BH_ | β | P | P_BH_ |
| rs7780562 | 0.037 | **0.008** | 0.240 | 0.032 | **0.031** | 0.690 | 0.051 | 0.233 | 0.981 |
| rs2954027 | -0.028 | **0.044** | 0.660 | -0.023 | 0.115 | 0.690 | -0.057 | 0.180 | 0.981 |
| rs10987829 | -0.023 | 0.093 | 0.683 | -0.025 | 0.079 | 0.690 | -0.015 | 0.720 | 0.981 |
| rs7412 | -0.023 | 0.095 | 0.683 | -0.024 | 0.1 | 0.690 | -0.006 | 0.896 | 0.981 |
| rs41280378 | -0.019 | 0.173 | 0.683 | -0.014 | 0.326 | 0.836 | -0.032 | 0.454 | 0.981 |
| rs6129772 | -0.019 | 0.175 | 0.683 | -0.023 | 0.113 | 0.690 | 0.014 | 0.746 | 0.981 |
| rs13306194 | -0.017 | 0.223 | 0.683 | -0.012 | 0.403 | 0.836 | -0.075 | 0.083 | 0.981 |
| rs7703282 | -0.017 | 0.230 | 0.683 | -0.014 | 0.341 | 0.836 | -0.029 | 0.495 | 0.981 |
| rs62074055 | 0.014 | 0.301 | 0.683 | 0.011 | 0.448 | 0.836 | 0.042 | 0.324 | 0.981 |
| rs12162136 | 0.014 | 0.315 | 0.683 | 0.008 | 0.584 | 0.836 | 0.03 | 0.472 | 0.981 |
| rs76898656 | -0.014 | 0.317 | 0.683 | -0.019 | 0.193 | 0.836 | 0.021 | 0.620 | 0.981 |
| rs7140110 | 0.014 | 0.328 | 0.683 | 0.011 | 0.468 | 0.836 | 0.022 | 0.613 | 0.981 |
| rs2539981 | -0.013 | 0.336 | 0.683 | -0.011 | 0.433 | 0.836 | -0.011 | 0.803 | 0.981 |
| rs553427 | -0.013 | 0.337 | 0.683 | -0.010 | 0.499 | 0.836 | -0.037 | 0.389 | 0.981 |
| rs1501908 | 0.013 | 0.357 | 0.683 | 0.015 | 0.295 | 0.836 | 0.008 | 0.853 | 0.981 |
| rs3741297 | -0.012 | 0.398 | 0.683 | -0.013 | 0.353 | 0.836 | 0.001 | 0.984 | 0.984 |
| rs629301 | -0.012 | 0.401 | 0.683 | -0.006 | 0.668 | 0.836 | -0.027 | 0.527 | 0.981 |
| rs9376090 | -0.011 | 0.410 | 0.683 | -0.015 | 0.298 | 0.836 | 0.028 | 0.506 | 0.981 |
| rs2642438 | -0.009 | 0.521 | 0.765 | -0.007 | 0.654 | 0.836 | -0.04 | 0.343 | 0.981 |
| rs17145738 | 0.008 | 0.549 | 0.765 | 0.006 | 0.697 | 0.836 | 0.035 | 0.413 | 0.981 |
| rs2419607 | -0.008 | 0.553 | 0.765 | -0.012 | 0.42 | 0.836 | -0.002 | 0.972 | 0.984 |
| rs11601507 | -0.008 | 0.561 | 0.765 | -0.009 | 0.518 | 0.836 | 0.004 | 0.916 | 0.981 |
| rs12229026 | -0.007 | 0.627 | 0.818 | -0.003 | 0.824 | 0.883 | -0.034 | 0.427 | 0.981 |
| rs59379014 | 0.005 | 0.713 | 0.828 | 0.002 | 0.869 | 0.899 | -0.007 | 0.872 | 0.981 |
| rs1883025 | -0.005 | 0.726 | 0.828 | 0.006 | 0.694 | 0.836 | -0.052 | 0.221 | 0.981 |
| rs2738464 | -0.005 | 0.743 | 0.828 | -0.007 | 0.633 | 0.836 | -0.007 | 0.862 | 0.981 |
| rs112784971 | -0.004 | 0.745 | 0.828 | -0.006 | 0.695 | 0.836 | -0.01 | 0.805 | 0.981 |
| rs3093679 | -0.003 | 0.832 | 0.891 | -0.005 | 0.736 | 0.849 | 0.02 | 0.645 | 0.981 |
| rs6493583 | -0.002 | 0.867 | 0.897 | 0.004 | 0.812 | 0.883 | -0.034 | 0.448 | 0.981 |
| rs11066015 | -0.001 | 0.926 | 0.926 | 0.001 | 0.925 | 0.925 | 0.014 | 0.740 | 0.981 |

**P_BH_: P-values after multiple adjustments using the Benjamini-Hochberg method.**

**Total: Adjust for age, gender, BMI, smoker, drinker.**

**Men, women: Adjust for age, BMI, smoker, drinker.**

**Table S8 Linear regression of enrolled SNPs and CT90 in our database**

| SNP | total |  |  | men |  |  | women |  |  |
| --- | --- | --- | --- | --- | --- | --- | --- | --- | --- |
|  | β | P | P_BH_ | β | P | P_BH_ | β | P | P_BH_ |
| rs76898656 | -0.027 | 0.059 | 0.591 | -0.031 | **0.039** | 0.785 | -0.002 | 0.962 | 0.987 |
| rs9376090 | -0.025 | 0.078 | 0.591 | -0.027 | 0.068 | 0.785 | 0.001 | 0.987 | 0.987 |
| rs2954027 | -0.024 | 0.092 | 0.591 | -0.015 | 0.307 | 0.787 | -0.078 | 0.067 | 0.987 |
| rs2539981 | -0.023 | 0.099 | 0.591 | -0.022 | 0.142 | 0.785 | -0.029 | 0.498 | 0.987 |
| rs17145738 | -0.021 | 0.143 | 0.591 | -0.024 | 0.111 | 0.785 | -0.001 | 0.982 | 0.987 |
| rs1501908 | 0.02 | 0.156 | 0.591 | 0.012 | 0.405 | 0.787 | 0.091 | **0.032** | 0.96 |
| rs62074055 | 0.019 | 0.172 | 0.591 | 0.018 | 0.218 | 0.787 | 0.023 | 0.597 | 0.987 |
| rs7780562 | 0.019 | 0.184 | 0.591 | 0.012 | 0.410 | 0.787 | 0.048 | 0.267 | 0.987 |
| rs2738464 | -0.018 | 0.191 | 0.591 | -0.021 | 0.157 | 0.785 | -0.019 | 0.655 | 0.987 |
| rs2419607 | -0.018 | 0.197 | 0.591 | -0.017 | 0.256 | 0.787 | -0.039 | 0.364 | 0.987 |
| rs7703282 | -0.016 | 0.253 | 0.690 | -0.022 | 0.149 | 0.785 | 0.035 | 0.423 | 0.987 |
| rs7412 | -0.014 | 0.309 | 0.726 | -0.018 | 0.222 | 0.787 | 0.021 | 0.632 | 0.987 |
| rs59379014 | -0.014 | 0.332 | 0.726 | -0.014 | 0.369 | 0.787 | -0.047 | 0.302 | 0.987 |
| rs2642438 | -0.013 | 0.339 | 0.726 | -0.014 | 0.36 | 0.787 | -0.016 | 0.699 | 0.987 |
| rs41280378 | -0.012 | 0.392 | 0.784 | -0.011 | 0.464 | 0.787 | -0.006 | 0.879 | 0.987 |
| rs10987829 | -0.011 | 0.433 | 0.812 | -0.012 | 0.437 | 0.787 | -0.02 | 0.635 | 0.987 |
| rs7140110 | 0.01 | 0.490 | 0.849 | 0.009 | 0.550 | 0.868 | 0.014 | 0.752 | 0.987 |
| rs11601507 | 0.009 | 0.510 | 0.849 | 0.005 | 0.714 | 0.903 | 0.034 | 0.420 | 0.987 |
| rs13306194 | 0.009 | 0.538 | 0.849 | 0.012 | 0.419 | 0.787 | -0.02 | 0.652 | 0.987 |
| rs12229026 | -0.007 | 0.605 | 0.890 | -0.004 | 0.804 | 0.906 | -0.033 | 0.44 | 0.987 |
| rs12162136 | 0.007 | 0.623 | 0.890 | 0.011 | 0.472 | 0.787 | -0.045 | 0.285 | 0.987 |
| rs11066015 | -0.006 | 0.681 | 0.927 | 0.003 | 0.815 | 0.906 | -0.051 | 0.232 | 0.987 |
| rs3093679 | 0.005 | 0.711 | 0.927 | 0.005 | 0.722 | 0.903 | 0.01 | 0.811 | 0.987 |
| rs6493583 | 0.003 | 0.828 | 0.979 | 0.006 | 0.690 | 0.903 | -0.001 | 0.979 | 0.987 |
| rs6129772 | -0.002 | 0.892 | 0.979 | -0.003 | 0.856 | 0.917 | -0.004 | 0.923 | 0.987 |
| rs629301 | 0.002 | 0.897 | 0.979 | 0.007 | 0.615 | 0.884 | -0.027 | 0.521 | 0.987 |
| rs553427 | -0.002 | 0.912 | 0.979 | -0.007 | 0.619 | 0.884 | 0.053 | 0.223 | 0.987 |
| rs3741297 | 0.002 | 0.914 | 0.979 | 0.001 | 0.969 | 0.987 | 0.002 | 0.959 | 0.987 |
| rs112784971 | 0 | 0.994 | 0.997 | -0.004 | 0.806 | 0.906 | 0.01 | 0.807 | 0.987 |
| rs1883025 | 0 | 0.997 | 0.997 | 0 | 0.987 | 0.987 | 0.027 | 0.532 | 0.987 |

**P_BH_: P-values after multiple adjustments using the Benjamini-Hochberg method.**

**Total: Adjust for age, gender, BMI, smoker, drinker.**

**Men, women: Adjust for age, BMI, smoker, drinker.**

**Table S9 Linear regression of enrolled SNPs and LSpO2 in our database**

| SNP | total |  |  | men |  |  | women |  |  |
| --- | --- | --- | --- | --- | --- | --- | --- | --- | --- |
|  | β | P | P_BH_ | β | P | P_BH_ | β | P | P_BH_ |
| rs2419607 | 0.029 | **0.043** | 0.470 | 0.029 | 0.055 | 0.613 | 0.038 | 0.373 | 0.979 |
| rs10987829 | 0.028 | **0.044** | 0.470 | 0.033 | **0.028** | 0.613 | 0 | 0.991 | 0.991 |
| rs2954027 | 0.028 | **0.047** | 0.470 | 0.02 | 0.18 | 0.613 | 0.087 | **0.038** | 0.390 |
| rs2539981 | 0.021 | 0.131 | 0.676 | 0.015 | 0.323 | 0.613 | 0.07 | 0.100 | 0.750 |
| rs112784971 | -0.021 | 0.132 | 0.676 | -0.015 | 0.309 | 0.613 | -0.056 | 0.186 | 0.930 |
| rs2642438 | 0.019 | 0.175 | 0.676 | 0.019 | 0.219 | 0.613 | 0.028 | 0.507 | 0.979 |
| rs7780562 | -0.019 | 0.186 | 0.676 | -0.011 | 0.451 | 0.644 | -0.058 | 0.175 | 0.930 |
| rs1883025 | 0.018 | 0.203 | 0.676 | 0.017 | 0.266 | 0.613 | -0.003 | 0.946 | 0.979 |
| rs76898656 | 0.016 | 0.251 | 0.676 | 0.021 | 0.168 | 0.613 | -0.013 | 0.759 | 0.979 |
| rs62074055 | -0.016 | 0.259 | 0.676 | -0.017 | 0.255 | 0.613 | -0.003 | 0.936 | 0.979 |
| rs3093679 | 0.0.16 | 0.269 | 0.676 | 0.015 | 0.331 | 0.613 | 0.023 | 0.598 | 0.979 |
| rs6129772 | 0.014 | 0.325 | 0.676 | 0.015 | 0.309 | 0.613 | 0.009 | 0.829 | 0.979 |
| rs1501908 | -0.014 | 0.337 | 0.676 | -0.014 | 0.354 | 0.613 | -0.022 | 0.608 | 0.979 |
| rs7703282 | 0.014 | 0.348 | 0.676 | 0.015 | 0.340 | 0.613 | -0.006 | 0.885 | 0.979 |
| rs6493583 | -0.013 | 0.375 | 0.676 | -0.017 | 0.277 | 0.613 | 0.004 | 0.933 | 0.979 |
| rs11601507 | 0.012 | 0.400 | 0.676 | 0.012 | 0.409 | 0.614 | 0.008 | 0.846 | 0.979 |
| rs12229026 | -0.011 | 0.432 | 0.676 | -0.024 | 0.109 | 0.613 | 0.088 | 0.039 | 0.390 |
| rs11066015 | 0.011 | 0.454 | 0.676 | 0.007 | 0.655 | 0.833 | 0.018 | 0.667 | 0.979 |
| rs2738464 | 0.01 | 0.465 | 0.676 | 0.013 | 0.388 | 0.613 | 0.007 | 0.861 | 0.979 |
| rs17145738 | 0.01 | 0.466 | 0.676 | 0.022 | 0.134 | 0.613 | -0.095 | 0.025 | 0.390 |
| rs9376090 | 0.01 | 0.473 | 0.676 | 0.015 | 0.329 | 0.613 | -0.033 | 0.437 | 0.979 |
| rs3741297 | 0.009 | 0.530 | 0.715 | 0.01 | 0.488 | 0.665 | 0.004 | 0.933 | 0.979 |
| rs13306194 | -0.009 | 0.548 | 0.715 | -0.013 | 0.387 | 0.613 | 0.031 | 0.469 | 0.979 |
| rs12162136 | -0.005 | 0.732 | 0.915 | -0.006 | 0.699 | 0.833 | 0.028 | 0.515 | 0.979 |
| rs7412 | -0.003 | 0.820 | 0.966 | -0.004 | 0.791 | 0.833 | -0.004 | 0.921 | 0.979 |
| rs7140110 | 0.003 | 0.851 | 0.966 | 0.007 | 0.669 | 0.833 | -0.018 | 0.673 | 0.979 |
| rs59379014 | -0.002 | 0.884 | 0.966 | -0.004 | 0.787 | 0.833 | 0.038 | 0.395 | 0.979 |
| rs629301 | 0.002 | 0.902 | 0.966 | -0.004 | 0.805 | 0.833 | 0.026 | 0.535 | 0.979 |
| rs41280378 | 0.001 | 0.965 | 0.967 | 0 | 0.985 | 0.985 | -0.013 | 0.767 | 0.979 |
| rs553427 | 0.001 | 0.967 | 0.967 | 0.004 | 0.782 | 0.833 | -0.03 | 0.488 | 0.979 |

**P_BH_: P-values after multiple adjustments using the Benjamini-Hochberg method.**

**Total: Adjust for age, gender, BMI, smoker, drinker.**

**Men, women: Adjust for age, BMI, smoker, drinker.**

**Table S10 Linear regression of enrolled SNPs and MAI in our database**

| SNP | total |  |  | men |  |  | women |  |  |
| --- | --- | --- | --- | --- | --- | --- | --- | --- | --- |
|  | β | P | P_BH_ | β | P | P_BH_ | β | P | P_BH_ |
| rs7140110 | 0.04 | **0.008** | 0.240 | 0.041 | **0.012** | 0.360 | 0.042 | 0.34 | 0.729 |
| rs7412 | -0.035 | **0.019** | 0.285 | -0.032 | **0.044** | 0.660 | -0.058 | 0.188 | 0.627 |
| rs2539981 | -0.029 | 0.050 | 0.500 | -0.025 | 0.118 | 0.968 | -0.073 | 0.093 | 0.626 |
| rs41280378 | -0.021 | 0.168 | 0.922 | -0.017 | 0.295 | 0.968 | -0.043 | 0.328 | 0.729 |
| rs7703282 | -0.019 | 0.200 | 0.922 | -0.019 | 0.244 | 0.968 | -0.018 | 0.692 | 0.952 |
| rs1883025 | -0.018 | 0.237 | 0.922 | -0.016 | 0.320 | 0.968 | -0.007 | 0.870 | 0.952 |
| rs629301 | -0.016 | 0.291 | 0.922 | -0.014 | 0.387 | 0.968 | -0.021 | 0.636 | 0.952 |
| rs11601507 | -0.013 | 0.387 | 0.922 | -0.013 | 0.403 | 0.968 | -0.012 | 0.791 | 0.952 |
| rs2738464 | -0.013 | 0.394 | 0.922 | -0.003 | 0.856 | 0.968 | -0.104 | **0.017** | 0.390 |
| rs3741297 | -0.012 | 0.425 | 0.922 | -0.004 | 0.776 | 0.968 | -0.082 | 0.059 | 0.590 |
| rs76898656 | -0.011 | 0.461 | 0.922 | -0.016 | 0.302 | 0.968 | 0.025 | 0.562 | 0.952 |
| rs12162136 | 0.009 | 0.543 | 0.922 | 0.012 | 0.450 | 0.968 | -0.032 | 0.462 | 0.924 |
| rs9376090 | -0.009 | 0.558 | 0.922 | 0 | 0.982 | 0.982 | -0.068 | 0.119 | 0.626 |
| rs10987829 | -0.008 | 0.591 | 0.922 | -0.011 | 0.485 | 0.968 | 0.008 | 0.856 | 0.952 |
| rs2954027 | -0.008 | 0.600 | 0.922 | -0.005 | 0.760 | 0.968 | -0.026 | 0.544 | 0.952 |
| rs12229026 | 0.008 | 0.600 | 0.922 | 0.011 | 0.501 | 0.968 | -0.006 | 0.889 | 0.952 |
| rs3093679 | -0.006 | 0.675 | 0.922 | -0.005 | 0.753 | 0.968 | -0.021 | 0.640 | 0.952 |
| rs6493583 | 0.006 | 0.715 | 0.922 | 0.003 | 0.869 | 0.968 | 0.046 | 0.322 | 0.729 |
| rs7780562 | -0.005 | 0.735 | 0.922 | -0.016 | 0.318 | 0.968 | 0.061 | 0.167 | 0.626 |
| rs11066015 | 0.005 | 0.737 | 0.922 | -0.001 | 0.936 | 0.968 | 0.066 | 0.128 | 0.626 |
| rs13306194 | 0.005 | 0.738 | 0.922 | -0.001 | 0.931 | 0.968 | 0.054 | 0.220 | 0.952 |
| rs62074055 | 0.005 | 0.738 | 0.922 | 0.003 | 0.852 | 0.968 | 0.009 | 0.834 | 0.660 |
| rs553427 | -0.005 | 0.761 | 0.922 | -0.008 | 0.636 | 0.968 | 0.014 | 0.749 | 0.969 |
| rs59379014 | -0.005 | 0.761 | 0.922 | -0.007 | 0.683 | 0.968 | 0.002 | 0.969 | 0.952 |
| rs1501908 | 0.004 | 0.768 | 0.922 | -0.001 | 0.933 | 0.968 | 0.061 | 0.158 | 0.626 |
| rs112784971 | -0.003 | 0.860 | 0.947 | -0.007 | 0.680 | 0.968 | 0.01 | 0.825 | 0.952 |
| rs6129772 | -0.002 | 0.874 | 0.947 | -0.002 | 0.892 | 0.968 | -0.005 | 0.912 | 0.952 |
| rs2419607 | -0.002 | 0.909 | 0.947 | 0.004 | 0.794 | 0.968 | -0.05 | 0.259 | 0.706 |
| rs2642438 | -0.001 | 0.944 | 0.947 | 0.012 | 0.453 | 0.968 | -0.097 | 0.026 | 0.390 |
| rs17145738 | -0.001 | 0.947 | 0.947 | -0.002 | 0.890 | 0.968 | -0.004 | 0.920 | 0.952 |

**P_BH_: P-values after multiple adjustments using the Benjamini-Hochberg method.**

**Total: Adjust for age, gender, BMI, smoker, drinker.**

**Men, women: Adjust for age, BMI, smoker, drinker.**

**Table S11 Linear regression of enrolled SNPs and glucose in our database**

| SNP | total |  |  | men |  |  | women |  |  |
| --- | --- | --- | --- | --- | --- | --- | --- | --- | --- |
|  | β | P | P_BH_ | β | P | P_BH_ | β | P | P_BH_ |
| rs7412 | 0.034 | **0.021** | 0.360 | 0.030 | 0.058 | 0.989 | 0.051 | 0.234 | 0.818 |
| rs59379014 | 0.034 | **0.024** | 0.360 | 0.047 | **0.003** | 0.989 | -0.012 | 0.779 | 0.695 |
| rs12162136 | 0.022 | 0.128 | 0.836 | 0.018 | 0.257 | 0.989 | 0.053 | 0.205 | 0.995 |
| rs3741297 | 0.022 | 0.131 | 0.836 | 0.028 | 0.070 | 0.998 | -0.026 | 0.538 | 0.818 |
| rs6493583 | 0.021 | 0.157 | 0.836 | 0.024 | 0.144 | 0.998 | 0.008 | 0.859 | 0.818 |
| rs7780562 | -0.02 | 0.170 | 0.836 | -0.009 | 0.585 | 0.998 | -0.072 | 0.091 | 0.818 |
| rs553427 | -0.019 | 0.195 | 0.836 | -0.022 | 0.157 | 0.989 | -0.016 | 0.705 | 0.818 |
| rs6129772 | 0.016 | 0.261 | 0.951 | 0.017 | 0.277 | 0.989 | 0.021 | 0.609 | 0.695 |
| rs2539981 | -0.014 | 0.327 | 0.951 | -0.004 | 0.810 | 0.989 | -0.095 | 0.023 | 0.818 |
| rs7140110 | -0.014 | 0.360 | 0.951 | -0.021 | 0.178 | 0.989 | 0.039 | 0.356 | 0.695 |
| rs76898656 | -0.012 | 0.388 | 0.951 | -0.005 | 0.736 | 0.989 | -0.052 | 0.214 | 0.987 |
| rs17145738 | 0.012 | 0.397 | 0.951 | 0.005 | 0.737 | 0.989 | 0.047 | 0.259 | 0.818 |
| rs11066015 | -0.011 | 0.426 | 0.951 | -0.021 | 0.169 | 0.989 | 0.032 | 0.436 | 0.695 |
| rs10987829 | 0.011 | 0.444 | 0.951 | 0.013 | 0.390 | 0.989 | 0.004 | 0.923 | 0.818 |
| rs11601507 | -0.01 | 0.503 | 0.962 | -0.032 | 0.039 | 0.998 | 0.103 | 0.013 | 0.695 |
| rs2419607 | -0.009 | 0.526 | 0.962 | 0.000 | 0.998 | 0.525 | -0.046 | 0.278 | 0.345 |
| rs2642438 | -0.007 | 0.609 | 0.962 | -0.013 | 0.399 | 0.989 | 0.029 | 0.481 | 0.987 |
| rs1883025 | -0.006 | 0.670 | 0.962 | 0.003 | 0.849 | 0.668 | -0.054 | 0.198 | 0.818 |
| rs2954027 | 0.006 | 0.688 | 0.962 | 0.010 | 0.516 | 0.989 | -0.019 | 0.652 | 0.695 |
| rs1501908 | -0.005 | 0.711 | 0.962 | -0.006 | 0.696 | 0.989 | -0.002 | 0.954 | 0.695 |
| rs9376090 | 0.005 | 0.751 | 0.962 | -0.003 | 0.857 | 0.668 | 0.053 | 0.204 | 0.818 |
| rs3093679 | 0.005 | 0.756 | 0.962 | 0.007 | 0.661 | 0.989 | -0.019 | 0.654 | 0.345 |
| rs7703282 | 0.004 | 0.767 | 0.962 | -0.004 | 0.802 | 0.831 | 0.051 | 0.232 | 0.818 |
| rs62074055 | 0.003 | 0.812 | 0.962 | 0.008 | 0.631 | 0.668 | -0.027 | 0.525 | 0.846 |
| rs112784971 | 0.003 | 0.813 | 0.962 | 0.000 | 0.991 | 0.989 | 0.019 | 0.643 | 0.695 |
| rs629301 | -0.002 | 0.880 | 0.962 | 0.000 | 0.977 | 0.668 | -0.024 | 0.563 | 0.954 |
| rs13306194 | 0.002 | 0.889 | 0.962 | 0.001 | 0.940 | 0.525 | 0.021 | 0.620 | 0.818 |
| rs41280378 | -0.002 | 0.916 | 0.962 | -0.004 | 0.820 | 0.831 | 0.000 | 0.995 | 0.695 |
| rs2738464 | -0.001 | 0.949 | 0.962 | -0.011 | 0.473 | 0.090 | 0.056 | 0.181 | 0.899 |
| rs12229026 | -0.001 | 0.962 | 0.962 | -0.004 | 0.817 | 0.525 | 0.020 | 0.636 | 0.695 |

**P_BH_: P-values after multiple adjustments using the Benjamini-Hochberg method.**

**Total: Adjust for age, gender, BMI, smoker, drinker.**

**Men, women: Adjust for age, BMI, smoker, drinker.**

**Table S12 Linear regression of enrolled SNPs and insulin in our database**

| SNP | total |  |  | men |  |  | women |  |  |
| --- | --- | --- | --- | --- | --- | --- | --- | --- | --- |
|  | β | P | P_BH_ | β | P | P_BH_ | β | P | P_BH_ |
| rs7780562 | 0.01 | **0.046** | 0.372 | 0.002 | 0.872 | 0.970 | 0.051 | 0.207 | 0.678 |
| rs41280378 | -0.026 | 0.054 | 0.372 | -0.027 | 0.067 | 0.503 | -0.012 | 0.763 | 0.921 |
| rs11066015 | 0.026 | 0.058 | 0.372 | 0.026 | 0.067 | 0.503 | 0.035 | 0.376 | 0.800 |
| rs3093679 | 0.026 | 0.058 | 0.372 | 0.023 | 0.118 | 0.536 | 0.057 | 0.161 | 0.678 |
| rs1501908 | -0.025 | 0.062 | 0.372 | -0.029 | **0.041** | 0.503 | 0.008 | 0.837 | 0.921 |
| rs3741297 | 0.023 | 0.082 | 0.377 | 0.011 | 0.452 | 0.969 | 0.113 | **0.005** | 0.150 |
| rs59379014 | 0.024 | 0.088 | 0.377 | 0.021 | 0.161 | 0.604 | 0.02 | 0.638 | 0.903 |
| rs2738464 | 0.022 | 0.105 | 0.394 | 0.013 | 0.353 | 0.883 | 0.073 | 0.068 | 0.510 |
| rs629301 | 0.02 | 0.143 | 0.465 | 0.022 | 0.125 | 0.536 | 0.013 | 0.745 | 0.921 |
| rs12229026 | 0.019 | 0.155 | 0.465 | 0.029 | 0.042 | 0.503 | -0.053 | 0.187 | 0.678 |
| rs11601507 | 0.018 | 0.174 | 0.475 | 0.015 | 0.282 | 0.791 | 0.036 | 0.368 | 0.800 |
| rs7140110 | -0.016 | 0.255 | 0.638 | -0.023 | 0.119 | 0.536 | 0.029 | 0.480 | 0.800 |
| rs6493583 | -0.015 | 0.282 | 0.651 | -0.008 | 0.613 | 0.970 | -0.06 | 0.151 | 0.678 |
| rs2539981 | -0.011 | 0.396 | 0.849 | -0.008 | 0.577 | 0.970 | -0.031 | 0.436 | 0.800 |
| rs7703282 | -0.01 | 0.455 | 0.882 | -0.015 | 0.290 | 0.791 | 0.032 | 0.424 | 0.800 |
| rs13306194 | -0.01 | 0.472 | 0.882 | -0.004 | 0.785 | 0.970 | -0.051 | 0.205 | 0.678 |
| rs2954027 | 0.009 | 0.500 | 0.882 | 0.008 | 0.574 | 0.970 | 0.024 | 0.543 | 0.857 |
| rs553427 | -0.008 | 0.556 | 0.891 | -0.012 | 0.398 | 0.918 | 0.036 | 0.379 | 0.800 |
| rs9376090 | -0.007 | 0.593 | 0.891 | -0.005 | 0.753 | 0.970 | -0.017 | 0.662 | 0.903 |
| rs6129772 | 0.007 | 0.610 | 0.891 | 0.018 | 0.216 | 0.720 | -0.077 | 0.053 | 0.510 |
| rs112784971 | 0.005 | 0.706 | 0.891 | -0.007 | 0.618 | 0.970 | 0.081 | 0.041 | 0.510 |
| rs17145738 | 0.005 | 0.732 | 0.891 | 0.003 | 0.825 | 0.970 | 0.007 | 0.863 | 0.921 |
| rs10987829 | 0.004 | 0.771 | 0.891 | 0.007 | 0.637 | 0.970 | -0.028 | 0.479 | 0.800 |
| rs7412 | 0.004 | 0.778 | 0.891 | 0.001 | 0.959 | 0.990 | 0.032 | 0.432 | 0.800 |
| rs1883025 | -0.004 | 0.790 | 0.891 | 0 | 0.990 | 0.990 | -0.007 | 0.861 | 0.921 |
| rs2642438 | -0.004 | 0.795 | 0.891 | -0.005 | 0.718 | 0.970 | 0.004 | 0.921 | 0.921 |
| rs2419607 | 0.003 | 0.802 | 0.891 | 0.003 | 0.827 | 0.970 | -0.005 | 0.893 | 0.921 |
| rs76898656 | 0.003 | 0.836 | 0.896 | 0.002 | 0.905 | 0.970 | 0.01 | 0.797 | 0.921 |
| rs12162136 | -0.002 | 0.873 | 0.903 | 0.002 | 0.882 | 0.970 | -0.048 | 0.226 | 0.678 |
| rs62074055 | 0 | 0.985 | 0.985 | 0.002 | 0.888 | 0.970 | -0.02 | 0.614 | 0.903 |

**P_BH_: P-values after multiple adjustments using the Benjamini-Hochberg method.**

**Total: Adjust for age, gender, BMI, smoker, drinker.**

**Men, women: Adjust for age, BMI, smoker, drinker.**

**Table S13 Linear regression of enrolled SNPs and HOMAIR in our database**

| SNP | total |  |  | men |  |  | women |  |  |
| --- | --- | --- | --- | --- | --- | --- | --- | --- | --- |
|  | β | P | P_BH_ | β | P | P_BH_ | β | P | P_BH_ |
| rs3741297 | 0.03 | **0.028** | 0.507 | 0.02 | 0.174 | 0.932 | 0.097 | **0.017** | 0.913 |
| rs59379014 | 0.029 | **0.042** | 0.507 | 0.032 | **0.035** | 0.932 | -0.003 | 0.935 | 0.830 |
| rs7412 | 0.025 | 0.068 | 0.507 | 0.023 | 0.119 | 0.932 | 0.045 | 0.277 | 0.913 |
| rs7140110 | -0.026 | 0.069 | 0.507 | -0.033 | **0.026** | 0.870 | 0.026 | 0.533 | 0.325 |
| rs1501908 | -0.023 | 0.095 | 0.507 | -0.027 | 0.068 | 0.932 | 0.008 | 0.837 | 0.888 |
| rs2738464 | 0.022 | 0.115 | 0.507 | 0.013 | 0.375 | 0.932 | 0.075 | 0.064 | 0.913 |
| rs3093679 | 0.022 | 0.122 | 0.507 | 0.018 | 0.222 | 0.932 | 0.049 | 0.24 | 0.888 |
| rs41280378 | -0.021 | 0.139 | 0.507 | -0.022 | 0.139 | 0.932 | -0.008 | 0.852 | 0.830 |
| rs629301 | 0.02 | 0.152 | 0.507 | 0.022 | 0.135 | 0.932 | 0.008 | 0.843 | 0.726 |
| rs11066015 | 0.015 | 0.264 | 0.792 | 0.011 | 0.431 | 0.690 | 0.048 | 0.236 | 0.325 |
| rs11601507 | 0.014 | 0.324 | 0.864 | 0.002 | 0.907 | 0.932 | 0.089 | 0.028 | 0.990 |
| rs12229026 | 0.013 | 0.351 | 0.864 | 0.02 | 0.170 | 0.932 | -0.034 | 0.411 | 0.325 |
| rs1883025 | -0.011 | 0.412 | 0.864 | -0.006 | 0.685 | 0.874 | -0.039 | 0.344 | 0.913 |
| rs553427 | -0.011 | 0.415 | 0.864 | -0.014 | 0.345 | 0.932 | 0.011 | 0.797 | 0.913 |
| rs2954027 | 0.011 | 0.432 | 0.864 | 0.011 | 0.442 | 0.932 | 0.014 | 0.737 | 0.643 |
| rs2539981 | -0.01 | 0.462 | 0.866 | -0.004 | 0.795 | 0.874 | -0.059 | 0.150 | 0.913 |
| rs17145738 | 0.009 | 0.511 | 0.902 | 0.008 | 0.584 | 0.870 | 0.012 | 0.771 | 0.913 |
| rs10987829 | 0.007 | 0.592 | 0.918 | 0.011 | 0.466 | 0.932 | -0.022 | 0.595 | 0.830 |
| rs112784971 | 0.007 | 0.603 | 0.918 | -0.006 | 0.704 | 0.653 | 0.081 | 0.046 | 0.830 |
| rs12162136 | 0.007 | 0.612 | 0.918 | 0.007 | 0.632 | 0.932 | 0 | 0.990 | 0.325 |
| rs6129772 | 0.005 | 0.703 | 0.966 | 0.018 | 0.230 | 0.874 | -0.081 | 0.047 | 0.726 |
| rs2419607 | -0.003 | 0.807 | 0.966 | 0.002 | 0.881 | 0.653 | -0.049 | 0.242 | 0.913 |
| rs6493583 | -0.003 | 0.807 | 0.966 | 0.002 | 0.873 | 0.653 | -0.036 | 0.401 | 0.913 |
| rs13306194 | 0.003 | 0.851 | 0.966 | 0.007 | 0.622 | 0.690 | -0.027 | 0.508 | 0.726 |
| rs62074055 | 0.002 | 0.871 | 0.966 | 0.004 | 0.781 | 0.870 | -0.015 | 0.715 | 0.325 |
| rs7780562 | 0.002 | 0.881 | 0.966 | -0.003 | 0.818 | 0.653 | 0.028 | 0.507 | 0.913 |
| rs7703282 | -0.002 | 0.887 | 0.966 | -0.013 | 0.377 | 0.525 | 0.076 | 0.065 | 0.888 |
| rs9376090 | -0.002 | 0.902 | 0.966 | -0.002 | 0.884 | 0.653 | 0.01 | 0.811 | 0.755 |
| rs2642438 | -0.001 | 0.939 | 0.967 | -0.006 | 0.685 | 0.525 | 0.033 | 0.415 | 0.967 |
| rs76898656 | -0.001 | 0.967 | 0.967 | 0.001 | 0.932 | 0.653 | -0.015 | 0.716 | 0.325 |

**P_BH_: P-values after multiple adjustments using the Benjamini-Hochberg method.**

**Total: Adjust for age, gender, BMI, smoker, drinker.**

**Men, women: Adjust for age, BMI, smoker, drinker.**
